# Feature Engineering and Supervised Machine Learning to Forecast Biogas Production during Municipal Anaerobic Co-Digestion

**DOI:** 10.1021/acsestengg.3c00435

**Published:** 2023-12-28

**Authors:** Hunter W. Schroer, Craig L. Just

**Affiliations:** † IIHR – Hydroscience and Engineering, University of Iowa, Iowa City, Iowa 52242, United States; ‡ Department of Civil & Environmental Engineering, University of Iowa, Iowa City, Iowa 52242, United States

**Keywords:** biomethane, anaerobic digestion, digital twin, SCADA, food waste

## Abstract

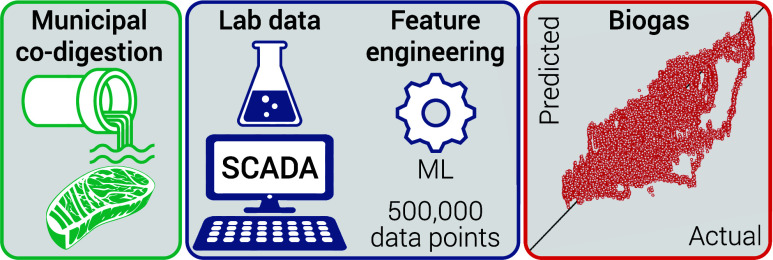

Municipalities with excess anaerobic digestion capacity accept offsite wastes for co-digestion to meet sustainability goals and create more biogas. Despite the benefits inherent to co-digestion, the temporal and compositional heterogeneity of external waste streams creates operational challenges that lead to upsets or conservative co-digestion. Given the complex microbial bioprocesses occurring during anaerobic digestion, prediction and modeling of the outcomes can be challenging, and machine learning has the potential to improve understanding and control of co-digestion processes. Biogas flows are a surrogate for process health, and here, we predicted biogas production from historical data collected by a water resource recovery facility (WRRF) during normal operation. We tested a daily lab and operational data set (*n* = 1089 after cleaning) and a minute-by-minute supervisory control and data acquisition (SCADA) operational data set (*n* = 491,761 after cleaning) to determine if forecasting biogas flow for a 24 h time horizon is feasible without collecting additional data. We found that a multilayer perceptron (MLP) neural network model outperformed tree-based and multiple linear regression models. Using a high-resolution SCADA data set for the first time, we showed that MLP neural networks could predict biogas production with an adjusted coefficient of determination (*R*^2^) of 0.78 and a mean absolute percentage error of 13.4% on a holdout test set. Adding daily laboratory analyses to the model did not appreciably improve the prediction of biogas flows. Feature engineering was essential to an accurate prediction, and 11 of the 15 most important features in the SCADA model were calculated from raw SCADA outputs. In summary, this paper demonstrates that minute-scale SCADA information collected at a municipal co-digestion facility can forecast biogas production, as a first step toward a digital twin model, without additional data collection.

## 1 Introduction

Anaerobic digestion (AD) is a mature technology that can turn organic wastes into valuable biogas, diverting organics from landfills and producing methane that can be used onsite or upgraded into renewable natural gas.^1^ Despite the ubiquitous and long-standing use of AD to treat municipal organic waste, the process still has practical difficulties due to the complexity of the heterogeneous microbial consortia and substrates involved.^2^ As climate change intensifies, AD of organics will become an increasingly important global technology to decrease greenhouse gas emissions and strengthen the circular economy, especially in the context of alternative substrates, including food waste, industrial organics, etc., that have historically been landfilled instead of digested for energy recovery.^1−3^ For example, savvy municipalities are accepting underutilized industrial organic wastes, collecting disposal fees, generating methane for heat and renewable energy, and meeting ambitious sustainability goals after potential odor and traffic concerns have been addressed by local residents. In fact, an estimated 20% of wastewater plants in the United States “co-digest” offsite wastes.^4^

Despite the inherent benefits and widespread adoption of co-digestion, the new feedstocks can create operational and nutrient discharge challenges that are uncommon for digestion of traditional municipal waste streams (e.g., primary sludge and waste-activated sludge).^1^ Due to the heterogeneity in composition and high loading rates common to co-digestion, the process can lead to costly downtime through digester foaming, upsets, and complete digester failure.^3^ Facilities may also need to consider side-stream nutrient treatment strategies when accepting additional organic loads. Due to the new challenges and opportunities that arise from anaerobic co-digestion at municipal WRRFs, new strategies to control and optimize biogas production are needed. Fortunately, WRRFs collect substantial operational data, and most have automated SCADA systems to synchronize sensor readings and process monitoring in real time.

As a first step toward understanding co-digestion dynamics and drivers, biogas flows are a reasonable surrogate for process health, are readily available for most facilities, and provide a straightforward target for modeling and prediction. A few studies have set out to predict biogas flows during co-digestion using machine learning (ML) models. One study explored partial dependence of biogas flows on different co-digestion substrates using an automated ML (AutoML) pipeline for a municipal facility to give substrate-by-substrate yield per ton estimates using daily data.^5^ However, this study used a random subsample for training and testing time-series data, which introduces data leakage and yields an overly optimistic model.^6^ Another study predicting biogas flows up to 40 days in advance compared different ML models but used an incorrect formula for testing coefficient of determination (*R*^2^) in their code.^7^ This minor mistake artificially inflated the *R*^2^ and resulted in a questionable model interpretation, as this is the only model performance metric discussed. One study used the SCADA system to monitor a traditional municipal AD plant on a daily time scale and artificial neural network (ANN) models to identify key operational parameters for biogas production with a small sample size.^8^ Another study used ML models to predict methane yield in laboratory reactors.^9^ A recent paper utilized AutoML pipelines to predict and explain biogas production from daily data from a dry fermentation system.^10^ Another study used a lab-scale system with online sensors to collect hourly data for 116 days and fit the data with deep learning models for predicting various AD parameters.^11^ Other studies that used ML models to predict biogas production were lacking a sufficient number of samples (<250) for accurate or generalizable performance, and are reviewed elsewhere.^5,12^ In summary, small sample sizes, controlled laboratory-scale systems, and some fundamental errors have resulted in studies that are difficult to use for comparison here. There is one paper that we highlight as applicable that used deep learning models on daily co-digestion data from a full-scale facility to predict biogas flow.^13^

Here, we demonstrate the first forecasting models for biogas production from co-digestion using data from a sub-hourly municipal SCADA system, providing the highest resolution data to date and a sample size 175 times greater than the next largest co-digestion data set.^5^ By comparing the minute-scale SCADA data with daily data over the same period, we demonstrate that little value is added from laboratory analysis of the waste streams, and the finer time scale and larger quantity of the SCADA system data provide a better opportunity for predicting biogas flows at this facility. We employed strict data leakage management, including time-ordered training and testing for time-series data, lagging biochemical oxygen demand by 5 days, and lagging daily operational totals and laboratory analyses so that they are appropriately available for prediction in real time. Our goal is to build a contemporaneous digital twin for the municipal WRRF to utilize for operational decision making, and this study is a proof of concept for using SCADA information for biogas prediction at a full-scale co-digestion facility.

## 2 Materials and Methods

### 2.1 Data Set Background and Preprocessing

#### 2.1.1 Facility Information

**Figure 1:**
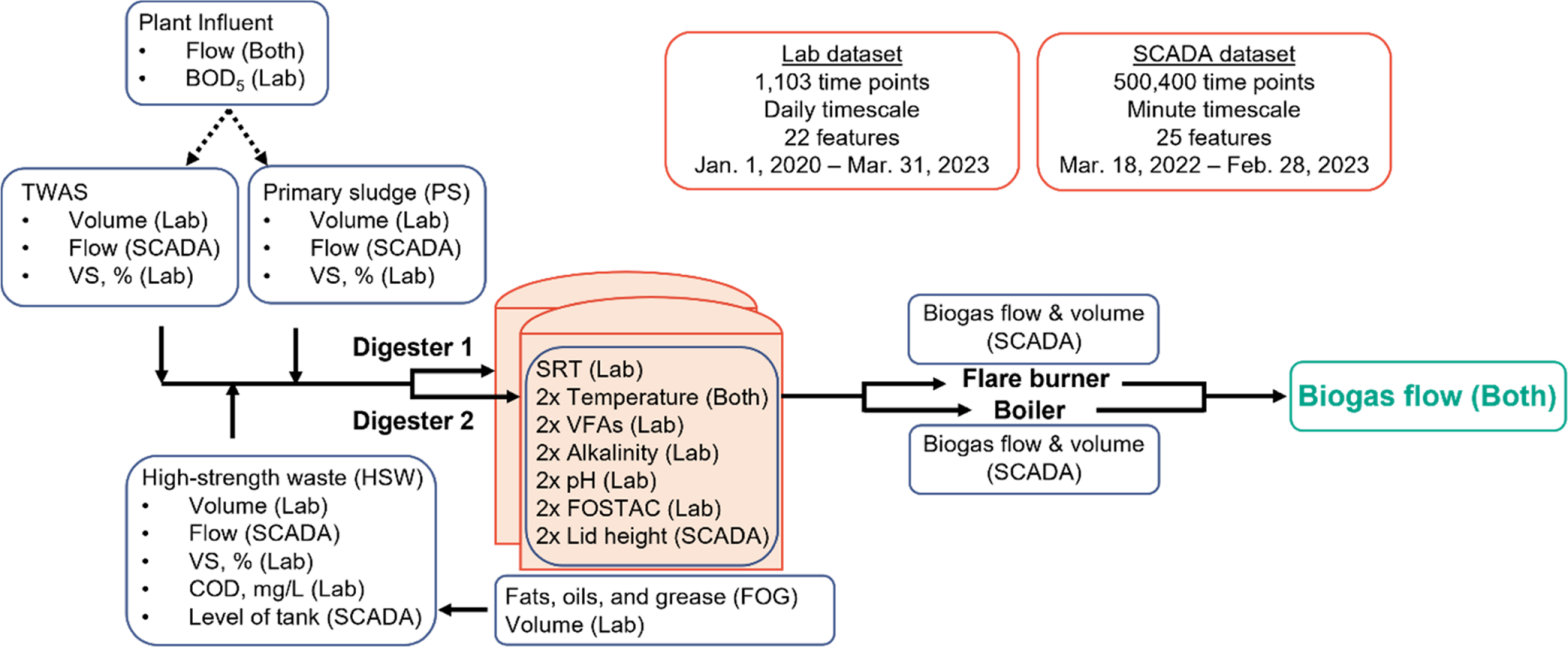
City of Muscatine Water Resource Recovery Facility anaerobic digester process flow diagram, relevant data inputs, and associated data set(s). Abbreviations: BOD_5_ = 5-day biochemical oxygen demand, TWAS = thickened waste activated sludge, VS = volatile solids, COD = chemical oxygen demand, mg/L = milligrams per liter, SRT = solids retention time, VFAs = volatile fatty acids, FOSTAC = ratio of VFAs to alkalinity, SCADA = supervisory control and data acquisition.

The City of Muscatine, Iowa WRRF is a 5.5 million gallons per day (MGD) treatment capacity plant that accepts industrial organic wastes for anaerobic co-digestion. The facility operates two 485,000 gallon continuously stirred tank reactors for mesophilic (36 °C) AD of sludge from wastewater treatment (after primary settling [PS] and after thickening of waste activated sludge by dissolved air flotation [TWAS]). The WRRF also receives fats, oils, and grease (FOG) from local commercial sources, which are added to a single, continuously mixed 65,000-gallon high-strength waste (HSW) tank. The HSW tank also receives organic waste streams from local food and feed processors (e.g., Kraft Heinz, Nestle Purina) after de-packaging, and the continuously mixed HSW is dosed into the digesters along with the PS and TWAS. The average PS and TWAS sludge volumes added to the digesters during the lab data collection period were 16,000 and 10,000 gallons per day, while the HSW flow averaged 13,000 gallons per day after HSW addition was initiated on March 16, 2020. Due to the highly variable HSW composition and quantity, the WRRF frequently encounters excessive foaming and digester upsets. To build biogas forecasting models, we utilized two data sets from the Muscatine WRRF: the first was collected daily by plant staff over 3 years, and the second was collected automatically as part of the facility’s SCADA system (Figure 1).

#### 2.1.2 Lab and Operational Data

The first data set was provided by staff at the City of Muscatine WRRF and includes daily totals for some process parameters or lab analyses from January 2020 to April 2023 for the entire facility. We selected a subset of all of the operational data for this study based on the potential impact on AD. Selected variables (and units) included: total daily volume (gallons) and volatile solids content (% weight per weight) of TWAS, PS, and HSW; chemical oxygen demand of the HSW (mg L^–1^); volume of FOG (gallons); solids retention time (days); plant influent flow (million gallons per day); plant influent biological oxygen demand (mg L^–1^); and, for each digester, temperature (°F), pH (standard units), alkalinity (mg CaCO_3_ L^–1^), volatile fatty acids (mg L^–1^), ratio of volatile fatty acids to alkalinity (mg VFA mg CaCO_3_^–1^). To prevent data leakage, we shifted biochemical oxygen demand (BOD) backward 5 days (i.e., BOD information is not available for at least 5 days from the current sampling date). Daily total biogas volume was recorded in cubic feet, and we converted the daily total to a daily average flow in cubic feet per minute by dividing by 1440 min per day. We excluded the period from April 6 to June 27, 2021, because the biogas flow meter was out of operation.

Next, we processed the data to fill in missing or suspect values as follows: We removed volatile fatty acid (VFA) analysis prior to April 21, 2020, due to a change in VFA laboratory analysis by WRRF staff from centrifugation supernatant to whole samples on this date. We removed weekend values for volatile solids (VS) and chemical oxygen demand (COD) that had been duplicated from weekday values (i.e., WRRF staff analyzed samples only on weekdays, but frequently copied Friday’s values to Saturday and Sunday). During the initial period from January 1, 2020, to March 15, 2020, the WRRF was not co-digesting HSW. Therefore, we set the VS and COD values for the HSW to zero so that these two parameters did not contribute artificially to biogas production values during modeling. Next, we set the values for HSW VS and COD for March 16, 2020 (when co-digestion began) to March 31, 2020, as equal to the values on April 1, 2020, which was the first date of measurement. We removed four values that appeared to be typos because no operational anomalies were documented by WRRF staff and the adjacent values were in the expected range: one digester 1 temperature (65.2 °F, 4/4/20), one digester 2 temperature (67.8, 8/30/22), one digester 2 pH (4.26, 10/12/20), and one digester 1 VFA (12,018 mg L^–1^, 8/11/20). We then populated any missing volume values as zero (i.e., no entry meant no volume was added) and filled missing values for other parameters using linear interpolation between available data points, assuming that other operational values (e.g., COD, pH, and VFAs) changed incrementally over time between measurements. The resulting data set contains 22 features, which are summarized in Table 1 and Figures S1 and 1.

**Table 1:** Summary of Raw Features in the “Lab” Data Set after Removing Four Presumed Data Entry Errors, as Described in Section 2^a^

variable	unit	min	max	mean	median	std. dev.	*n*
biogas flow	CFM	1.4	236.8	103.5	101.3	41.5	1103
V, TWAS	gallons	213	29,985	10,176	9205	4630	1103
V, PS	gallons	249	229,022	16,451	15,998	11,513	1103
V, HSW	gallons	0	50,237	12,372	11,188	8886	1103
V, FOG	gallons	0	56,055	12,350	12,423	11,024	1103
VS, TWAS	%	0.30	4.85	3.09	3.12	0.53	1046
VS, PS	%	0.33	6.54	3.01	2.95	0.85	725
VS, HSW	%	0.86	22.16	6.88	6.60	3.01	654
COD, HSW	mg/L	4457	1,123,025	150,600	133,000	86,575	529
SRT	days	3.6	165	24.7	22.2	11.0	1103
T, dig. 1	°F	85.0	98.5	95.5	95.6	0.9	1098
T, dig. 2	°F	85.0	126.1	95.6	95.7	1.6	1102
pH, dig. 1	S.U.	6.43	7.94	7.25	7.27	0.2	1093
pH, dig. 2	S.U.	6.49	7.76	7.21	7.23	0.1	1094
alkalinity, dig. 1	mg/L	1965	8610	5039	5043	1124	960
alkalinity, dig. 2	mg/L	2222	8565	4994	4985	1070	959
VFAs, dig. 1	mg/L	191	3635	1,276	1,178	582	861
VFAs, dig. 2	mg/L	257	4114	1,274	1,135	628	861
FOSTAC, dig. 1	unitless	0.11	0.93	0.25	0.23	0.09	861
FOSTAC, dig. 2	unitless	0.10	0.80	0.25	0.23	0.10	861
Q, influent	MGD	1.93	7.98	3.38	3.11	0.92	1103
BOD, influent	mg/L	46.2	1870	490	450	250	1101

a Std. dev., standard deviation; CFM, cubic feet per minute; V, volume; TWAS, thickened waste activated sludge; PS, primary sludge; HSW, high-strength waste; FOG, fats, oils, and grease; VS, volatile solids; COD, chemical oxygen demand; mg/L, milligrams per liter; SRT, solids retention time; T, temperature; Dig., digester; F, Fahrenheit; S.U., standard units; VFAs, volatile fatty acids; FOSTAC, ratio of volatile fatty acids to alkalinity; Q, flow; BOD, biochemical oxygen demand.

We next used domain knowledge for feature selection and feature engineering on the data set. We removed the “volume of FOG” variable to eliminate double counting because the FOG is mixed into and accounted for by the HSW volume. We assumed that composite digester health would be a stronger predictor of biogas flow than any individual variable and that correlated operational parameters would artificially reduce their individual contributions to a forecasting model. Accordingly, we created a new feature called “digester stability” that incorporated temperature, pH, alkalinity, VFAs, and ratio of VFAs to alkalinity for both digesters following the conceptual framework of a previous “digester stability” score,^14^ and excluded the 10 original variables (full calculation details are provided in Text S1). Next, we noticed that biogas flow had a weekly periodic pattern (Figure S2), so we added a variable for “day of the week” for later transformation into a cyclical variable with sine and cosine pairs. We postulated that a combined VS load would be more important than either flow or VS percentage for each influent constituent and multiplied flow and VS percent variables into three “VS load” variables (TWAS, PS, and HSW) and removed the individual VS and flow variables. Similarly, we calculated the HSW COD load as the product of HSW COD and HSW flow and subsequently removed the original HSW concentration. Although HSW COD and HSW VS loads were highly correlated (0.86 by Spearman rank sum), we used empirical model-based analysis, rather than *a priori* knowledge, for eliminating HSW COD load later, as described below.

To account for the effect of time-series data, we added duplicate, time-lagged features to some variables. We included six additional variables (lagged 1–6 days, respectively) for HSW VS load, HSW COD load, PS VS load, and TWAS VS load. We also added six lagged variables for the biogas flow. For prediction, we added the “forecast” variable as a new vector, with “forecast” at time *t* equal to “biogas” at time *t*–*h*, where *h* is the forecast horizon. Note that for model implementation and use, the daily totals would only be known the following day, and a forecast of 1 day is, at best, a prediction of the current day’s daily average biogas flow. Finally, we removed rows without forecast or lagged-variable data prior to further analysis, resulting in 1089 time points and 39 features for a one-day forecast.

#### 2.1.3 SCADA Information

The second data set analyzed in this work consisted of 25 variables, selected for relevance to the AD process, recorded by the Muscatine WRRF SCADA system on a minute time scale from March 18, 2022, to February 28, 2023 (500,884 observations). We manually identified 484 rows with zeroes for multiple parameters (assumed to be power surges) and calculated summary statistics (Table 2), and then temporarily replaced all columns in these rows with interpolated values as placeholders. Next, we calculated the biogas flow variable by first summing the flow to the waste gas burner and flow to the boiler. Then, to match the lab data set “Biogas” total flow variable, we calculated the rolling average total biogas flow over the previous 24 h. Finally, we added the “forecast” variable (i.e., target variable for forecasting models) as a new vector with “forecast” at time *t* equal to rolling average total biogas flow over the previous 24 h at time *t*–*d*, where *d* is the forecast horizon.

**Table 2:** Summary of Raw Variables Selected from the SCADA Data Set^a^

variable	unit	minimum	maximum	mean	median	std. dev.
Q, biogas to burner	CFM	0.0	212.2	84.0	92.0	47.1
Q, biogas to boiler	CFM	0.0	120.0	28.8	28.3	28.7
V, boiler biogas today	ft^3^	0	97,751	21,568	17,646	16,728
V, burner biogas today	ft^3^	0	100,000	52,890	49,990	34,638
Q, primary sludge	MGD	0.00	500.00	11.28	0.08	58.11
Q, influent	MGD	0.00	24.07	3.24	3.11	1.55
Q, TWAS	GPM	0.11	120.97	7.65	0.19	19.96
Q, HSW to dig. 1	GPM	0	60.00	6.65	2.63	8.48
Q, HSW to dig. 2	GPM	0	60.00	3.94	0.00	6.84
V, HSW (total, dig. 2 yesterday)	ft^3^	0	31,409	5611	4069	5490
H, HSW tank	ft	0.66	13.72	4.98	4.95	1.00
H, dig. 1 lid	ft	0.11	7.40	6.01	5.97	0.58
H, dig. 2 lid	ft	2.87	6.50	4.46	4.33	0.44
T, dig. 1	°F	85.0	101.9	96.0	96.1	0.85
T, dig. 2	°F	85.0	126.9	96.1	96.1	1.85

a Number of observations is 500,400 after power surges were removed manually. Std. dev., standard deviation; Q, flow; CFM, cubic feet per minute; V, volume; ft^3^, cubic feet; MGD, million gallons per day; TWAS, thickened waste activated sludge; GPM, gallons per minute; HSW, high-strength waste; Dig., digester; H, height; ft, feet; T, temperature; F, Fahrenheit.

We then conducted further feature selection and engineering to minimize the number of unimportant variables and increase forecasting ability. First, we removed variables that were redundant or unlikely to affect biogas flows, including aeration basin air flow, recycled activated sludge flows and controls, and waste activated sludge flow to the dissolved aeration flotation treatment system, as the sludge flows to the digesters are accounted for in the TWAS and PS flows. We combined HSW flow to each digester into one variable for total HSW flow and added four cyclical variables using a sine and cosine transformation on the unit-circle-normalized hour of the day and day of the week, respectively. We noticed that the “cumulative volume of biogas to the burner today” variable frequently maxed out at 100,000 cubic feet, so we recalculated the variable as the number of minutes since midnight times the average burner gas flow rate from midnight to the current time (Figure S3). We created an average flow for the past 24 h for the TWAS, PS, and HSW flows and removed the volume of HSW to digester 2 yesterday because digester 2 had periods where it was offline and received no HSW. We created a variable that was the derivative of the digester lid height in units of ft min^–1^ by taking the difference in the current value and the value 1 min prior. We set the lid height derivatives for time “2022-08-24 14:09” to zero (median value) because the calculated derivatives were artificially high at this time due to an unexplainable difference in lid heights. We created a variable that was the average total biogas flow for the previous nominal hour (e.g., at times 08:00 to 08:59, the “Biogas_prev_hour_avg” variable is the average of the total flow from 07:00 to 07:59). Next, we added variables that were average flows of PS, TWAS, HSW, and biogas over the previous 24 h, as calculated above, lagged in increments of 1 day up to 4 days (i.e., “Biogas_prev_24h_*i*” at time *t* is total biogas flow, averaged over the previous 24 h, at time *t-i* days). Finally, we removed rows with missing data due to time-shifted variables and then removed the 484 interpolated placeholder rows prior to further analysis. The resulting data set consisted of 491,761 observations (with a 1-day forecast) and 38 features.

#### 2.1.4 Combined Data Set

For the third data set, we combined the two original data sets to contain approximately 1 year of daily lab and operational data and minute-by-minute SCADA readings. To inform real-time prediction, the dates of the lab and operational data set were shifted to the prior day to prevent data leakage (i.e., daily totals and averages are only available for previous days). We also removed the biogas flow and lagged biogas flow variables from the lab data set and used the SCADA biogas values as the target for model prediction.

#### 2.1.5 Forecasting over Different Time Horizons

We tested the effect of forecast horizon on model performance using the best models for each data set, with the hyperparameters tuned to a 1-day forecast. We changed the forecast vector to match the corresponding horizon and deleted the rows that did not have forecast values due to missing data. The resulting data set sizes are listed in Table S1.

### 2.2 Forecasting Models

#### 2.2.1 Model Evaluation and Baseline “Persistence” Model Definition

Model evaluation was conducted on holdout test data sets. Ultimately, we selected the best model based on the Akaike Information Criterion (AIC) and minimum testing error index (TeEI), which includes the adjusted coefficient of determination (adj. *R*^2^), mean absolute percentage error (MAPE), and root mean squared error (RMSE), which were calculated using eqs 1–5^15^
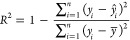
1
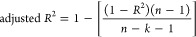
2
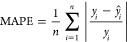
3
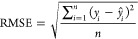
4
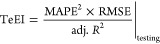
5
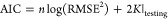
6where *n* is the number of observations, *k* is the number of features, and *K* is the number of model parameters.

As a baseline for model performance comparison, we defined a “persistence” model, in which the new prediction of biogas flow is simply the current value of biogas flow. For the lab data set, this was “tomorrow’s biogas flow equals today’s biogas flow.” For the SCADA and combined data sets, we used the engineered feature “average biogas flow over the previous nominal hour” as the prediction for the average biogas flow over the next 24 h.

#### 2.2.2 Feature Selection and Modeling with Multiple Linear Ridge Regression

We first forecasted biogas flow 1 day in advance from the three data sets using a ridge regression model, which is robust to multicollinearity among input variables.^16^ To prevent data leakage in time-series forecasting, it is essential to separate training, cross-validation, and testing data as a function of time, rather than using a random distribution of training and testing data.^6,17^ Accordingly, we selected a time-ordered training/testing split ratio of 0.8/0.2 based on visual inspection of histograms of the “forecast” biogas flow variable to approximately match the observations between the training and testing data sets (Figures S4 and S5), while still maintaining an appropriate number of samples in each category and a sufficient training sample-to-feature ratio (23 for lab and operational and 10,000 for SCADA, prior to additional feature selection).^6^ We standardized the data by scaling each variable to a mean of zero and a unit variance. Finally, we fit the model using ridge regression, with the optimal ridge penalty parameter selection with 5-fold cross-validation. We used the *TimeSeriesSplit* time-series cross-validator from the scikit-learn library, in which successive training sets are time-ordered supersets of previous training sets,^18^ with 5-fold cross-validation and a gap of 5 days for the lab and operational data set and 5 h for the SCADA data set.

We tested the effect of adding a periodic time-dependent variable that was fit to the day of the week. First, we assigned the day of the week an integer from 0 to 6 and then scaled the variable to the unit circle by dividing by 6 and multiplying by 2π to get *d*. Next, we tested 1–10 sine and cosine pairs (e.g., sine(*id*) and cosine(*id*) for *i* = 1–10) as new variables as described by Newhart et al.^17^ While the periodic model did improve the linear model predictions (data not shown), we ultimately decided to remove the daily periodic variables to include only deterministic variables that were independent of historical operations. That is, the historical biogas flow rate peaks on Thursday, but the trend is almost certainly due to the HSW feeding schedule (Figure S2). Therefore, instead of using the day of the week, we wanted to link models more mechanistically to waste flows rather than to this artificial predictor.

**Figure 2:**
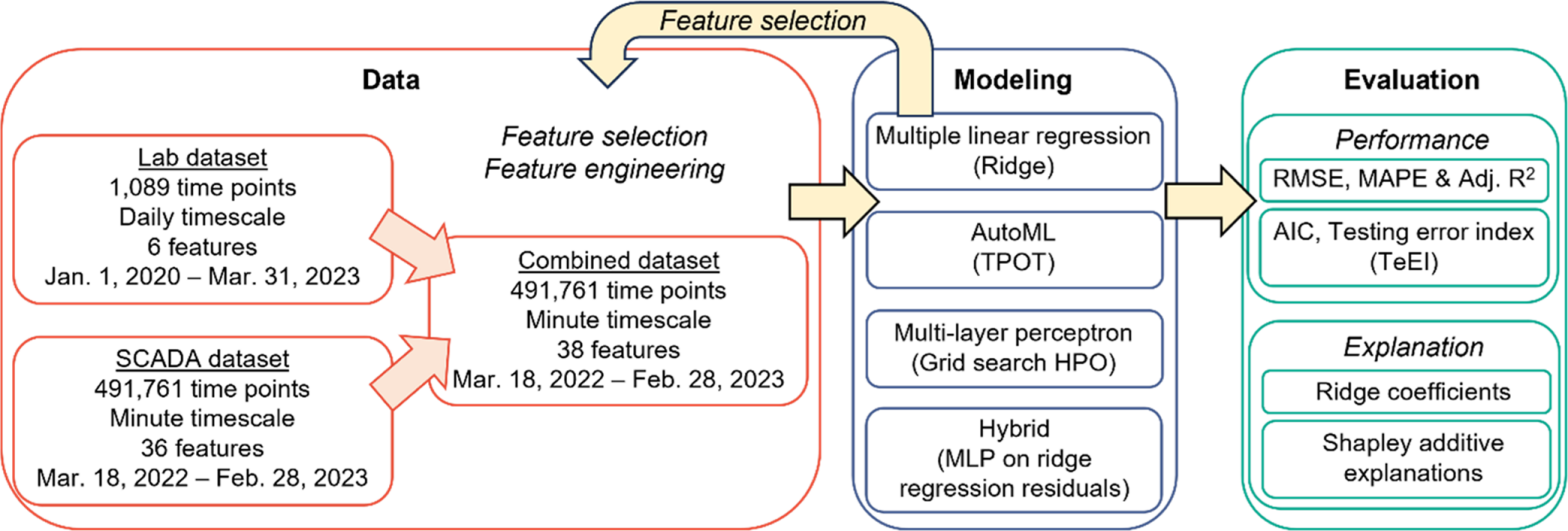
Conceptual diagram of the data, modeling, and evaluation presented in this paper.

We used Spearman rank sum correlation and statistical significance of linear model coefficients (α = 0.05) to eliminate features that were not important to the multiple linear regression model. For the daily lab data set, HSW COD load and HSW VS load were highly correlated (0.86 by Spearman rank sum correlation), and we removed HSW COD load (and lagged HSW COD loads) because the combined coefficients were smaller than those for HSW VS load and lagged HSW VS loads. Next, using backward elimination,^19^ we sequentially removed variables by testing the significance of all variable coefficients from a ridge regression, removing the single variable with the highest *p*-value, and then refitting the model until all variable coefficients had a *p*-value less than the test criteria (α = 0.05). The resulting variables were used for ridge regression, tree-based ML, and neural network models, as described below (Figure 2). We calculated the coefficient *p*-values using the *stats* function in Python *Regressors* package. We calculated and visualized the Spearman rank sum correlations using the Python *Seaborn* library.

We also compared the feature sets selected by the ridge regression method to an adaptive lasso feature selection method using ridge regression for initial parameter estimation.^17^ Adaptive lasso yielded the same six features for the lab data set. Adaptive lasso yielded a sparser, five-variable subset (“Biogas_burner”, “Q-HSW_GPM”, “Biogas_prev_hour_avg”, “Biogas_prev_24h”, and “Q-HSW_GPM_prev_24h_2”) for the SCADA data set. However, despite the simplicity of the smaller feature set, the resulting multiple linear regression models and MLP had higher testing errors compared with the larger “ridge regression” feature set. Therefore, we proceeded with the richer feature set from the ridge regression feature selection method.

#### 2.2.3 Tree-Based Pipeline Optimization Tool

After initial multiple linear regression modeling was completed, we hypothesized that ML models could supplement or replace the interpretable, linear models to capture some of the nonlinear effects of the full-scale and dynamic biogas generation process. While the linear regression models have simple and understandable inputs, the co-digestion process likely contained nonlinearities that were not captured by simple models. ML models have the drawback that they provide no insight into mechanistic connections between independent variables and observations; however, they are more robust to co-correlated variables, such as those found in time-series problems such as this one and have been frequently applied for biogas prediction.^7,10,13^

We used an automated ML (AutoML) tool to create a robust ML pipeline without biases for model selection, feature standardization, and hyperparameter optimization. To do so, we used the Tree-based Pipeline Optimization Tool (*tpot* library in Python), which selects feature preprocessing operators, conducts feature selection, combines optimal models, and conducts hyperparameter optimization using genetic programming to compile an ML pipeline.^20^ We used the TPOT regressor with time-series cross-validation with the parameters described above. We set the scoring function to minimize the mean squared error, the population size to 100, and the generations to 500. We then used the output pipeline for downstream model evaluation without further parameter optimization or data pretreatment.

#### 2.2.4 Multilayer Perceptron

Next, to compare the tree-based AutoML models to neural network models that are not included in the TPOT algorithm, we utilized the multilayer perceptron regressor (*MLPRegressor* class from scikit learn). A multilayer perceptron (MLP) is a feed-forward ANN that trains based on backpropagation. The MLP in this case is made up of an input layer containing the same number of neurons as input variables: (a) hidden layer(s) that contains neurons, which each transform the input from the previous layer using a nonlinear activation function; and an output layer that receives the values from the final hidden layer and yields a continuous value.^18^ We standardized the data by scaling each variable to a mean of zero and unit variance and conducted hyperparameter tuning on the training sets using grid search and a 5-fold time-ordered cross-validation. We set the grid search to minimize mean absolute percentage error and search for optimum values for the number of hidden layers, number of hidden neurons, strength of the L2 regularization term (α), learning rate, and solver, with search parameters and results for each data set summarized in Table S2.

#### 2.2.5 Model Interpretation

Model interpretation is critical to uncover how each feature contributes to model predictions and to understanding model decision-making processes. In the multiple linear regression models, the coefficient of each variable is a straightforward indicator for importance. The magnitude and sign of the regression coefficients are directly proportional to the target prediction (for a uniformly scaled data set, as used here). In nonlinear ML models, such as the MLPs employed here, the interpretation is more complicated. To uncover variable contributions to our MLP models, we used Shapley additive explanations (SHAP) analysis (*shap* Python package), which calculates the contributions of each individual feature for each individual prediction (i.e., local explanation) but can also be generalized to the entire data set to provide global interpretation (i.e., global explanation).^21^ Higher absolute SHAP values over the entire model translate to higher model importance. Due to its ease of calculation and interpretation, SHAP values have been commonly used in other environmental studies.^6,10,22,23^ For our data sets, we first trained the SHAP kernel explainer on a summary of the training set based on 10 weighted *k*-means. For the lab data set, we then fit the explainer to the entire test set. For the SCADA and combined data sets, we fit the explainer to a random subset of 15,000 points from the respective test set to reduce computation time.

### 2.3 Computing Resources and Data Accessibility

We used Python 3.11 for all code and predominantly used a 32-core, 512 Gb computing node through the University of Iowa interactive data analytics service, with some larger operations (MLP grid searches, TPOT runs on the larger data sets) completed on the University of Iowa Argon high-performance computing cluster on 56- to 80-core, 256–312 Gb computing nodes. Code is available as an archived GitHub repository at doi.org/10.5281/zenodo.8360096. Data sets are archived in an open-source repository at doi.org/10.25820/data.006715.

## 3 Results and Discussion

### 3.1 Lab Data Set

The lab data set contained co-correlated features, specifically HSW loads of VS and COD. We removed HSW COD loads and additional features that did not have significant coefficients in the multiple linear regression model using backward elimination based on *p*-values of the coefficients (α = 0.05). The resulting feature set contained only six features for predicting tomorrow’s biogas flow: biogas total flows today, yesterday, and 5 days ago, and total HSW VS load today, yesterday, and 6 days ago (Table S3). It was intriguing but not surprising that only two variables and their corresponding lagged values were the best predictors of biogas flow, given the autocorrelation of biogas flow and HSW VS over time (Figure S6). As shown in Figure S1, of the three influent streams (PS, TWAS, and HSW), HSW had the highest variability in terms of flow and VS content. It was notable, however, that the linear models did not account for any variability in the PS and TWAS flow. Also notable was that despite the high similarity between the distribution of COD and VS loads in the HSW, COD was worse at predicting biogas flows as compared to VS (TeEI of 3.03 cubic feet per minute [CFM] excluding COD compared to TeEI of 3.29 CFM excluding VS in the ridge regression model).

**Figure 3:**
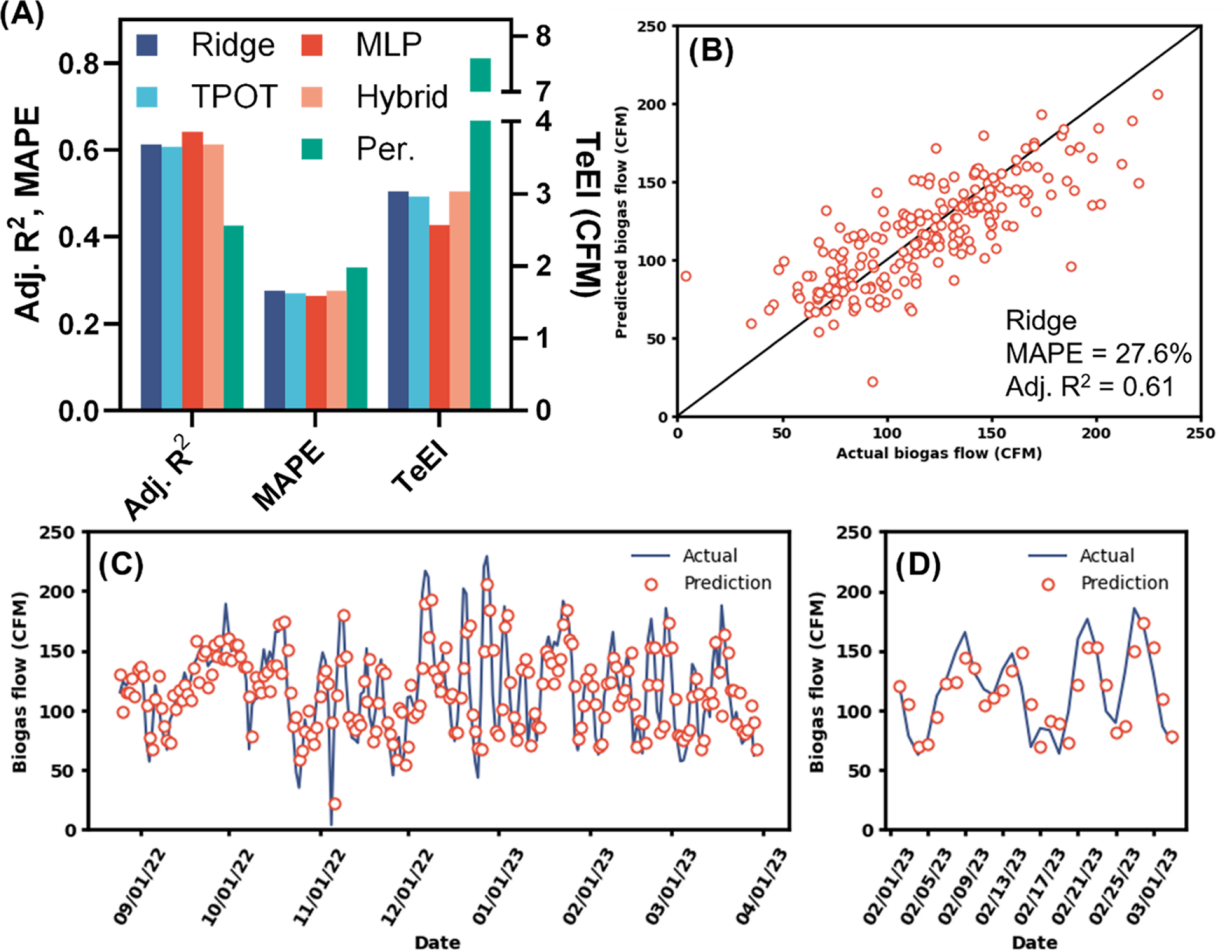
Lab data set. (A) Adjusted coefficient of determination (*R*^2^), mean absolute percentage error (MAPE), and testing error index (TeEI) of the test data set for each model evaluated. (B) Predicted vs actual biogas flows for the ridge regression model. (C) Testing time series of predicted and actual biogas flows for the ridge regression model. (D) Subset of plot (C). TPOT is a tree-based pipeline optimization tool. MLP, multilayer perceptron; Per., persistence; CFM, cubic feet per minute.

A ridge regression was able to predict the biogas flow with a MAPE of 27.6% and a TeEI of 3.03 CFM (Figure 3A). Next, we tested if ML models could outperform simple regression for predicting biogas flows, given the same variables used in the ridge regression models. Initial testing indicated that the feature subset selected from ridge regression coefficient significance was more optimal than the full feature set. Additionally, other recent environmental studies have used linear regression coefficients as a feature selection preprocessing step.^15,24,25^ Interestingly, the tree-based AutoML model (a feature scaling function followed by a linear support vector regression, see Text S1) barely outperformed the simple multiple linear regression (Figure 3A). We next fit the lab data with an MLP neural network regressor, which outperformed ridge and TPOT regressor models. Finally, we investigated a hybrid linear-MLP model, in which an MLP regressor was fit to error in the linear model prediction. Surprisingly, the MLP model on the residual error of the linear regression did not improve upon the linear regression model, whether or not the linear prediction value was included in the MLP training data (data including linear prediction not shown). For the lab data and models tested, the MLP model was the best-performing model in terms of testing error, with a testing MAPE of 26.5%, adjusted *R*^2^ of 0.64, and TeEI of 2.57 CFM. While the models could predict general trends in biogas flow (Figure 3C,D), the adjusted *R*^2^ of ∼0.6 was below the predictive ability of a similar literature study using daily biogas predictions^10^ and indicated that the causes for the wide daily swings in biogas production may not be captured by daily composite sampling and data collection.

**Figure 4:**
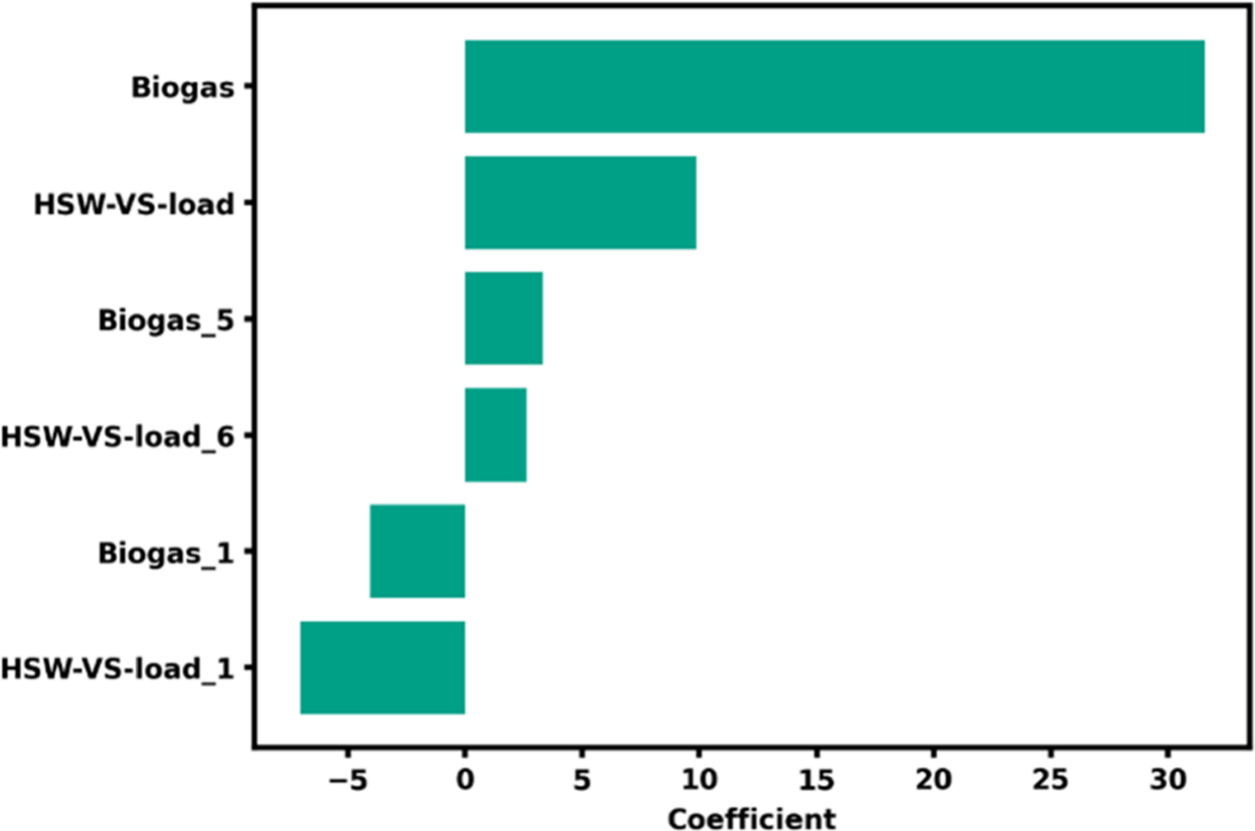
Coefficients for the lab multiple linear regression model.

Despite the best testing accuracy, the MLP model was more complex and less interpretable than the ridge regression model, and we selected the ridge regression model for further analysis based on a lower AIC score (Table S4). We then plotted the ridge regression coefficients to determine which features were most important to the model (Figure 4). Clearly, biogas today was the most important predictor for biogas tomorrow (Figure 4), which was not unexpected due to biogas flow autocorrelation (Figure S6). Despite the biogas flow today contributing the most information to the regression prediction, the baseline persistence (TeEI of 7.61 CFM) was easily outperformed by all of the models (Figure 3A). The VS load of HSW and the VS load of HSW lagged 1 day rounded out the top three most important (i.e., highest absolute value of coefficients) features in the lab ridge regression model. Given the autocorrelation of HSW VS (Figure S6), it was not surprising that the HSW VS load today and yesterday had opposite effects; i.e., if the load is high today, the load was likely high yesterday. The same effect was seen in the biogas flows, with biogas today and lagged 5 days contributing positively, and biogas flow lagged 1 day contributing negatively.

### 3.2 SCADA Data Set

**Figure 5:**
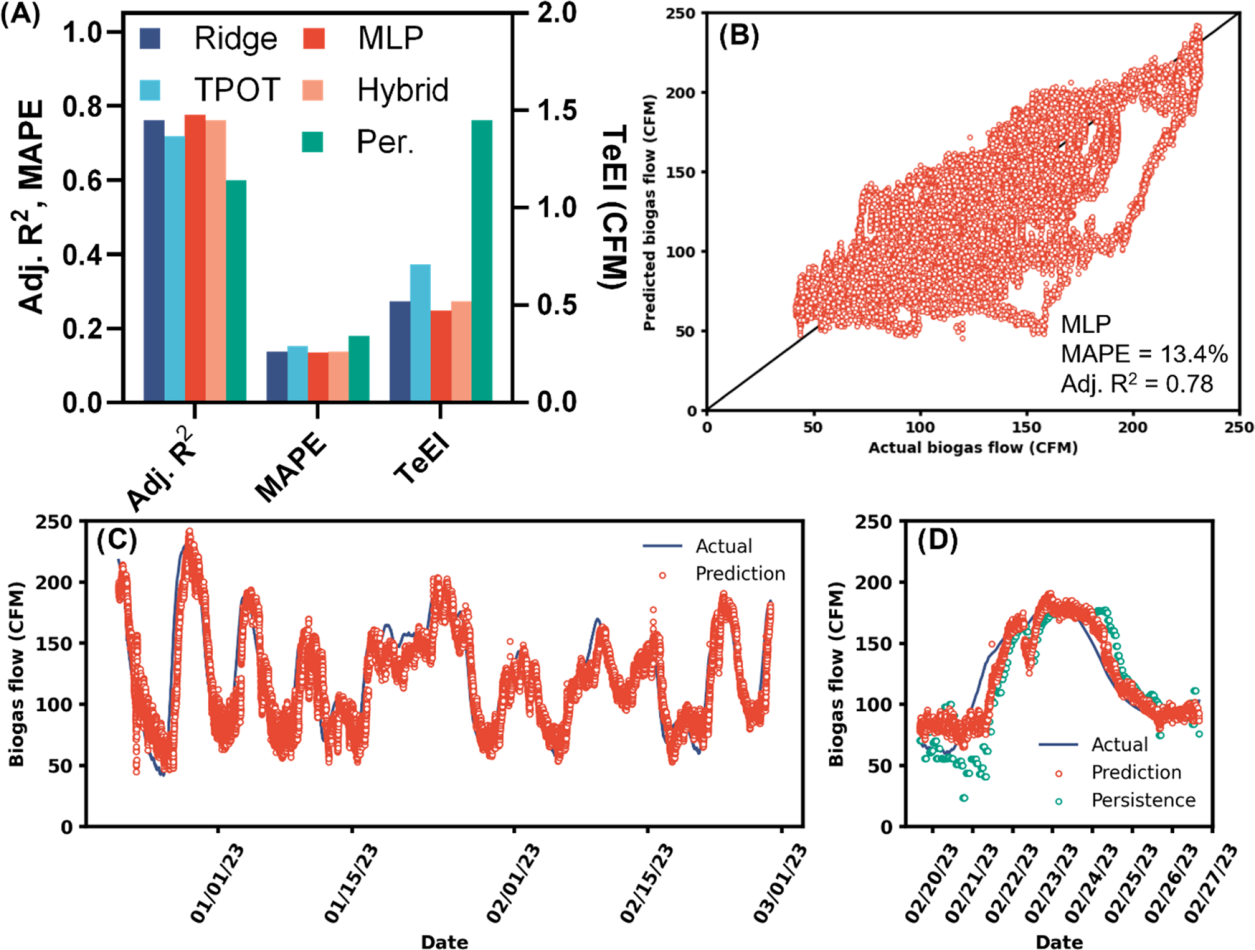
SCADA data set. (A) Adjusted coefficient of determination (*R*^2^), mean absolute percentage error (MAPE), and testing error index (TeEI) of the test data set for each model evaluated. (B) Predicted vs actual biogas flows for the MLP model (best-performing model). (C) Testing time series of predicted and actual biogas flows for the MLP model. (D) Subset of 1 week from plot (C) that also includes the persistence model (Per.). TPOT, tree-based pipeline optimization tool; CFM, cubic feet per minute.

For the SCADA data set, we eliminated digester 2 temperature and the plant influent flow because the coefficients in a multiple linear regression were not significant (α = 0.05). The resulting data set contained 36 features (Table S3). A ridge regression fit the data well (adjusted *R*^2^ = 0.76, TeEI = 0.52 CFM). Compared to the lab data set, the SCADA data set immediately had more potential for forecasting biogas flow. For example, the SCADA persistence model had a TeEI of 1.45 CFM, compared to 7.61 CFM for the lab data set. The ridge regression model had a MAPE of only 14%, compared to 28% for the lab data set (Figure 5). Clearly, the higher resolution of biogas flow in the SCADA data set (minute vs daily scale) increased prediction potential, despite the longer time period covered by the lab data set (3 years vs 1 year for SCADA).

Similar to the lab data set, a ridge regression outperformed the persistence model. Surprisingly, when the SCADA data set was fit with the TPOT AutoML pipeline (a stacked ensemble of linear regressors, see Text S2), the resulting model performed slightly worse than the simple ridge multiple linear regression model (Figure 5). As with the lab data set, the MLP model was the best model and achieved a TeEI of 0.47 CFM and a testing MAPE and adjusted *R*^2^ of 13.4% and 0.78, respectively. The MLP results were comparable to previous results from daily data modeled with a complicated deep learning ensemble (testing *R*^2^ = 0.76).^13^ A hybrid model with an MLP fit to the residuals of the linear model did not outperform the linear model. Despite its complexity, the MLP had a lower AIC value than the ridge regression model. Therefore, we selected the MLP model for further analysis.

During large changes in biogas flow over the scale of days, the MLP predictions lagged behind the actual biogas flow (Figure 5D). However, the MLP was able to match the changes better than the persistence model (biogas flow for the previous nominal hour, Figure 5D). The MLP model underpredicted the actual biogas flow with greater magnitude than the overpredictions (Figure 5B), which suggests that a sharp increase in biogas flow may be partly caused by factors not represented well by the features as compared with decreases in biogas flow. Due to the autocorrelation of biogas flows over time, there were periods of hours at a time where the model markedly underpredicted the actual biogas flow. This is likely because when the biogas flow increased sharply, the average flow over the previous hour, which was the most important variable, had not started to increase, so the model continuously predicted a low flow. In Figure 5D, we can see an example where biogas flow increased sharply on February 20, 2023, and the MLP model and persistence model were slow to correct for the actual flow prediction. When the flow started to decrease on February 24, 2023, the change is more gradual, and both the persistence and MLP model had more accurate predictions during a decrease in flow as compared to an increase.

**Figure 6:**
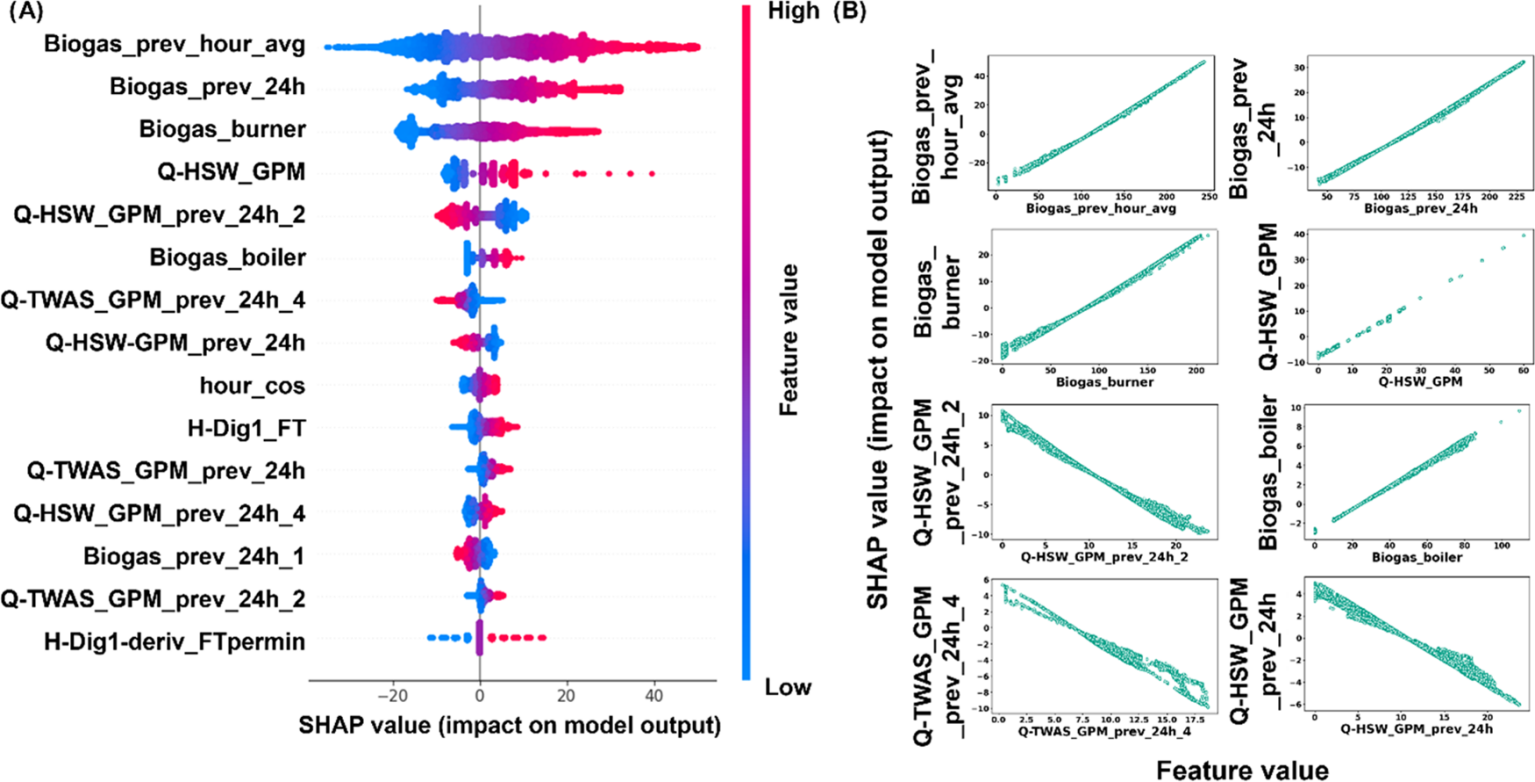
SCADA data set. (A) Shapely (SHAP) values as a function of feature value for each feature in the MLP model. (B) Individual dependence plots of the SHAP values as a function of the feature values for the MLP model for the eight most important variables.

We conducted a feature importance analysis on the MLP model using SHAP values (Figure 6). A larger SHAP value indicates a higher impact on model output, and the features are ranked in order of importance from top to bottom in Figure 6A. As shown in Figure 6, the most important variables for predicting biogas flow over the next 24 h were biogas flow the previous (nominal) hour, biogas flow over the previous 24 h, and biogas flow to the burner. Clearly, biogas flow the previous nominal hour was a critical engineered feature, both in terms of baseline prediction as a persistence model, where it much outperformed biogas flow the previous 24 h (Figure S7), and as a key variable in the forecasting models. The current flow of HSW and the 24 h average flow of HSW lagged 2 days rounded out the top five most important features in the MLP model (Figure 6). While the current HSW flow and top three biogas flow features were additive (more important to the model at higher feature values), the 24 h average flow of HSW lagged 2 days had the opposite effect and decreased the predicted biogas flow at high values. The HSW flow and its average and lagged average values indeed made up four of the top 15 important features. Engineered features were essential to the model. Indeed, 11 of the top 15 features in the SCADA data set were calculated from the raw data (i.e., engineered). In the dependence plots in Figure 6B, vertical dispersion or nonlinear patterns were a result of the nonlinear MLP model, and a multiple linear regression would have had perfectly linear plots for each variable. Generally, the top SCADA features were highly linear in the SHAP dependence plots (Figure 6B). Given the linearity of the features, a “deeper” learning MLP model with two hidden layers could not match the performance of a single hidden layer. Interestingly, biogas flow in the previous hour and in the previous 24 h were nearly linear. We began to see a small amount of nonlinearity in the HSW and TWAS flows, and the HSW flow was slightly less linear than the biogas flow, in general. Even though the dependences are close to linear, the small amount of variation from linear explains why the MLP model outperformed a ridge multiple linear regression.

### 3.3 Combined Data Set

**Figure 7:**
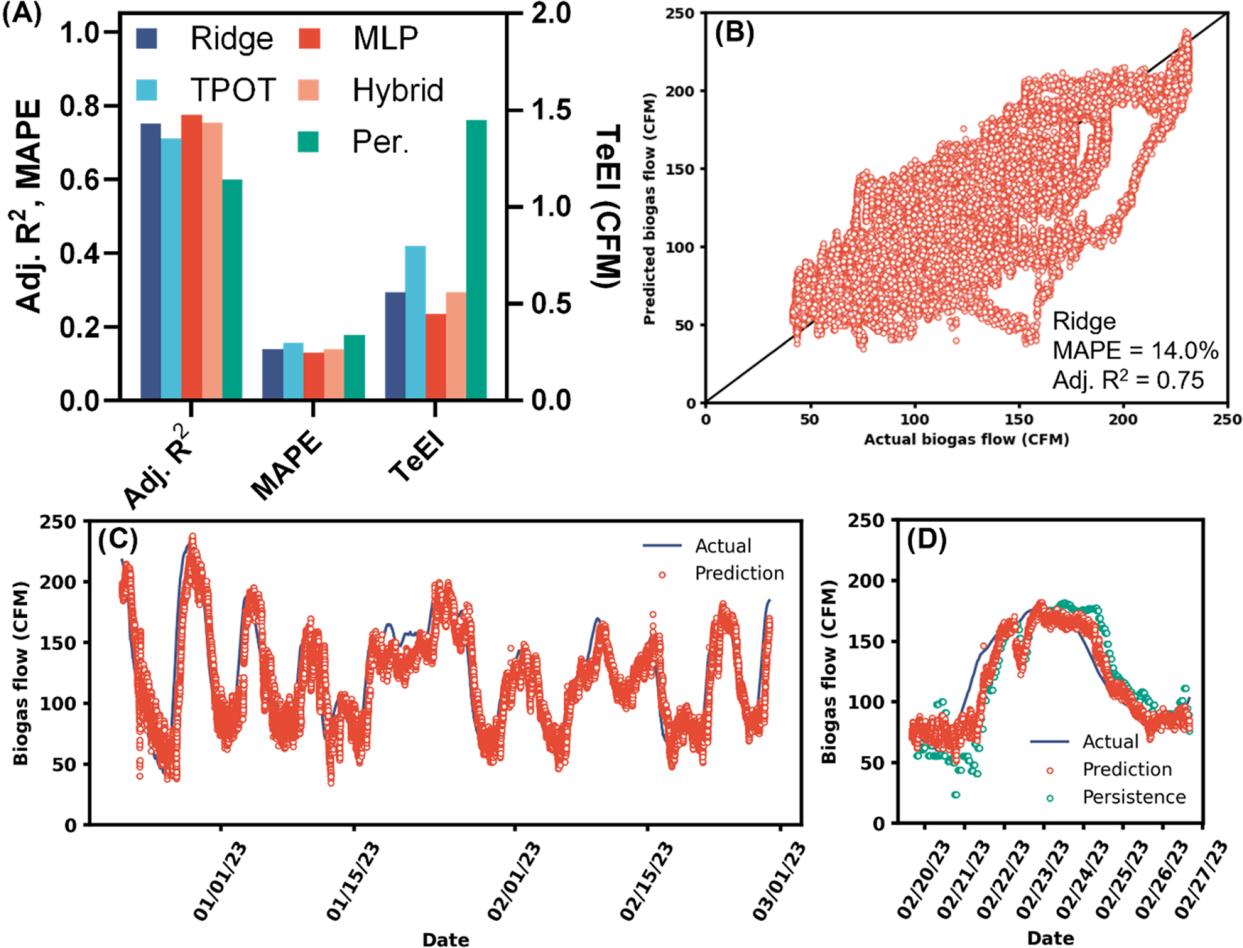
Combined data set. (A) Adjusted coefficient of determination (*R*^2^), mean absolute percentage error (MAPE), and testing error index (TeEI) of the test data set for each model evaluated. (B) Predicted vs actual biogas flows for the ridge multiple linear regression model. (C) Testing time series of predicted and actual biogas flows for the ridge regression model. (D) Subset of 1 week from plot (C) that also includes the persistence model (Per.). MLP, multilayer perceptron; TPOT, tree-based pipeline optimization tool; CFM, cubic feet per minute.

We hypothesized that adding laboratory compositional analysis of the HSW to the SCADA information would improve the biogas flow prediction. We tested adding all laboratory and SCADA features as a combined data set. We first removed biogas flows from the lab data set (we used the SCADA biogas flow as the model target) and then used backward elimination to remove variables as described above for each individual data set, and the resulting models surprisingly performed worse than the SCADA data set alone. We next tested the subsets of the best predictors from each individual data set by removing all features that were not significant in the individual data set models and then removing 24 h average PS flow lagged 2 days because it was not significant to a ridge regression model on the combined data set, which left 38 features (Table S3). This subset of best features from the individual data sets performed better than starting from the full feature set from both individual data sets. As with the individual data sets, a ridge multiple linear regression fit the data to an acceptable degree, achieving a MAPE of 14.0% and a TeEI of only 0.56 CFM, compared to a MAPE of 17.9% and a TeEI of 1.45 CFM for the persistence model (Figure 7A). To test tree-based ML model prediction, we ran the data set through the TPOT AutoML pipeline, and the resulting pipeline (feature selection and linear support vector regression) performed worse than the simple multiple linear regression, with a TeEI of 0.80 CFM. Next, we fit the data with an MLP model, which had the lowest testing error index, with a testing MAPE of 13.1%, adjusted *R*^2^ of 0.78, and a TeEI of 0.45 CFM.

Given the similarity of the combined data set to the SCADA data set (one variable removed and only three lab variables added), it was surprising to see the combined data set perform worse than the SCADA data set in the ridge regression (with all variables and with a subset of the best variables from each individual data set). Upon reexamination, we tested removing the SCADA HSW flows averaged over the previous 24 h and the same variable lagged 1 day (which likely contain similar information as the lab HSW VS load variables and had Spearman correlations of >0.7). A ridge regression performed slightly worse (TeEI of 0.57 CFM compared to 0.56 CFM with all variables) and the MLP model also saw a decrease in performance (TeEI of 0.51 CFM without “Q-HSW_GPM_prev_24h”, and “Q-HSW_GPM_prev_24h_1”). It should also be noted that the same MLP structure that was tuned for the SCADA data (one hidden layer of 20 neurons) performed worse on the combined data set than the SCADA data set, despite the high similarity between the two. The optimal MLP that minimized TeEI for the combined data set consisted of two hidden layers with 400 and 100 neurons, respectively, which took significantly longer to train when compared to the single hidden layer of the SCADA model and had similar performance. Based on a substantially smaller AIC when compared to the MLP model, we selected the ridge regression for further analysis (Figure 7B–D).

**Figure 8:**
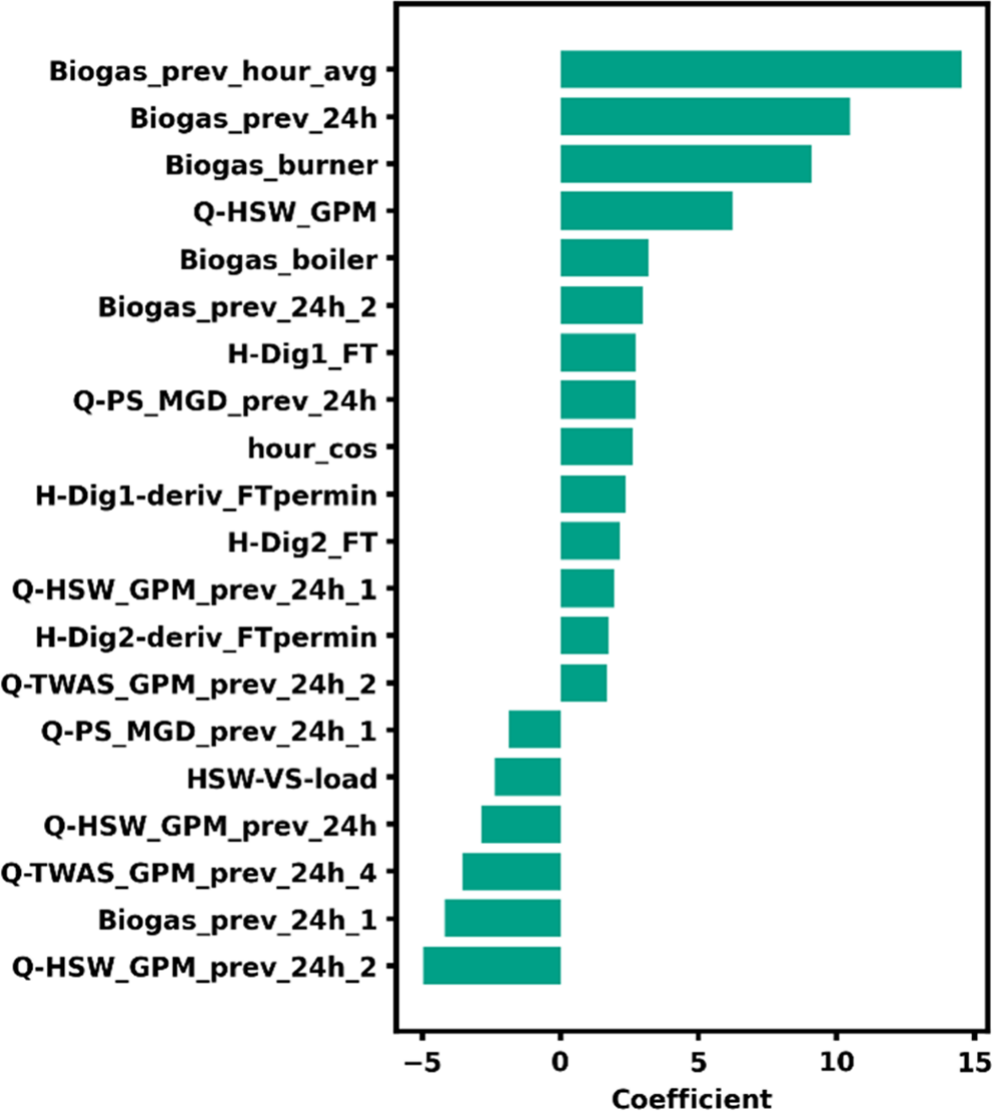
Coefficients of the 20 most important variables (i.e., highest absolute value) in the combined data set for a ridge multiple linear regression model.

We plotted the 20 ridge regression coefficients with the largest absolute values to determine which features were most important to the model (Figure 8). As with the SCADA model, biogas flow over the previous hour and the previous 24 h were the most important predictors for biogas flow tomorrow in the combined model. The most important features were similar between the SCADA MLP and the combined ridge regression (Figures 6 and 8). Perhaps the most important conclusion from the feature importance analysis was that the laboratory analysis of HSW VS content multiplied by HSW flow to produce HSW VS load in the lab data set was not important to the combined model. Of the lab data set variables added in the combined data set, the most important was HSW VS load lagged 1 day, which ranked 17 out of the 38 features. Therefore, the daily laboratory data did not aid in biogas flow forecasting, at least in terms of how the models and features were structured here.

### 3.4 Effect of Forecast Horizon on Model Performance

**Figure 9:**
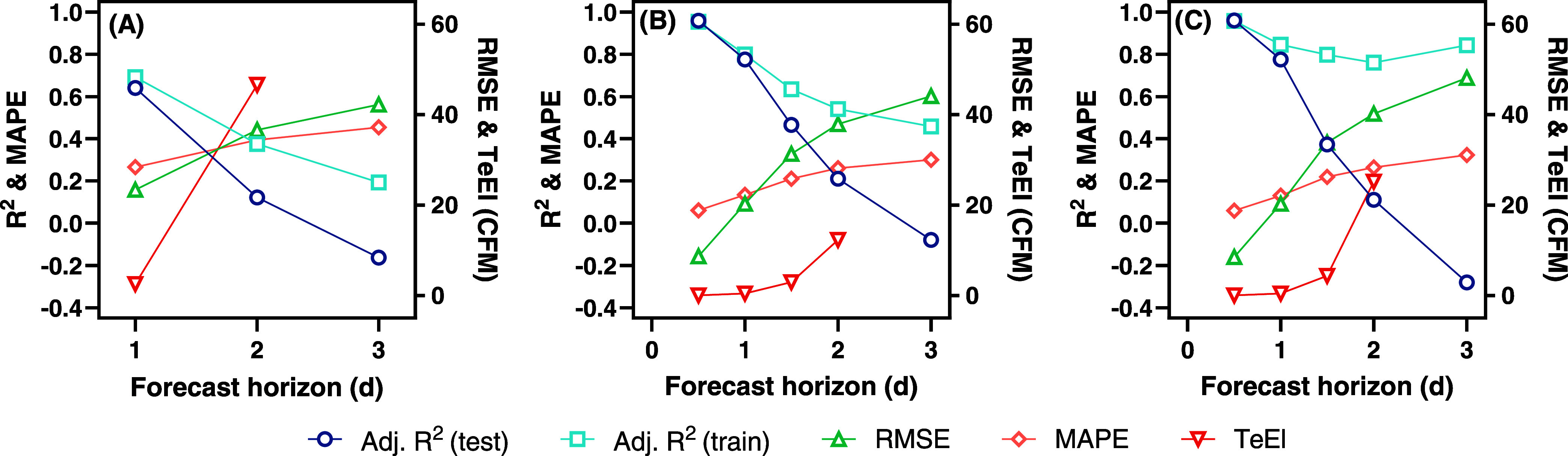
Model evaluation metrics for the best-performing MLP models as a function of forecast horizon for the (A) lab, (B) SCADA, and (C) combined data sets. Note that the TeEI is meaningless and is therefore not plotted, when *R*_test_^2^ < 0.

Clearly, each data set is useful for predicting biogas flows 1 day in advance. Prediction further in advance would be increasingly functional for feedstock selection and dosing, and we tested each model for a variety of forecast horizons (Figure 9). For all three data sets, model performance was best for 1 day or less. When extending to 1.5 or 2-day predictions, there was a large drop in model generalization as seen in the performance on the test data sets. The reader should note that model parameters were set to fixed values, and hyperparameters were tuned on the 1-day forecast. Better prediction at longer time horizons may be possible with hyperparameter tuning for the longer forecast horizon data sets or by using more complicated multistep predictions. It was also interesting to note the high training *R*^2^ that was maintained by the MLP fit to the combined data set. This suggests that the model was still able to learn important features from the combined data set, and better longer-term predictions may be possible with hyperparameter tuning for that specific case, though the 3-day model obviously had extreme overfitting.

## 4 Conclusions

Despite a few examples of previous work to predict biogas flows during anaerobic co-digestion, this is the first study to utilize real-time municipal plant data on a minute scale. We are actively working to incorporate our SCADA MLP model into a real-time, digital twin dashboard that can inform decision-making for operators at the Muscatine WRRF for feedstock selection and dosing. We hope to deliver a method for additional operational insight for the highly dynamic co-digestion system that could be applied to all co-digestion facilities with SCADA systems already collecting underutilized data and lower the barrier to co-digestion implementation.

The predictive models developed here demonstrate that the HSW composition is less important for predicting biogas flow than initially expected. Apparently, the waste and biogas flows are more predictive than the composition of HSW, despite the variability in HSW VS content. The HSW VS load data is also, by definition, “outdated” at prediction time (i.e., lab data is a daily average and only available to the model for the previous day to prevent data leakage) compared to current biogas and waste stream flows. For example, for a prediction at 23:00, the HSW VS load is assumed to be an average of the period from 23 to 47 h prior to the current time and was likely sampled for VS analysis the previous morning, around 40 h prior. Perhaps the actual variability in VS content is not captured well enough with VS analysis 5 days per week but could be improved with sampling at a higher frequency. Online sensors that can give real-time composition analysis of the waste streams would likely improve biogas prediction, and we are working to add near-infrared spectrometers to monitor the HSW influent and digester contents.

As this work represents a proof of concept for collecting and implementing municipal SCADA data for decision making in real time, there are many limitations and areas for improvement. Overall, all models tested (multiple linear regression, MLP, tree-based machine learning algorithms, and hybrid linear models with an MLP correcting the error) performed better than persistence, indicating that SCADA information coupled to fairly straightforward data smoothing and modeling could enable biogas flow prediction at other municipal facilities. However, as the SCADA system at each municipality collects different parameters and each WRRF has a unique process flow configuration, generalization of the model structures could be limited due to this specificity. An additional consideration for implementation at other facilities could be feedstock variability. Higher variability in feedstock quantity and composition at other facilities may present modeling issues and will require a case-by-case investigation. Generally applicable techniques include data leakage management for co-digestion time-series data, consideration of appropriate smoothing windows, prediction targets, and persistence models, a simple multiple linear regression method for feature selection (adaptive lasso or ridge), and the integration of high-resolution data, rather than daily composite sampling, if the process is highly dynamic. Future work will focus on improving predictive models by testing additional models, such as long short-term memory (a recurrent neural network), smoothing variables (especially biogas flow) on different rolling time scales to minimize noise for better prediction, trying different subsets of features with different feature selection strategies, and integrating the model into the Muscatine WRRF operational framework.

## Supporting Information Available

Additional text describing the digester stability calculation and the TPOT outputs; tables with additional information on model features, grid search parameters, and model evaluation results; and figures with summaries of the feature data, autocorrelation of features, and persistence model comparisons (PDF)
