# Supplementary material for: Feature Engineering and Supervised Machine Learning to Forecast Biogas Production during Municipal Anaerobic Co-Digestion
Source: ACS ES T Eng. 2023 Dec 28;4(3):660–72. doi: 10.1021/acsestengg.3c00435 (PMC10928704; doi:10.1021/acsestengg.3c00435)
Supplement: Supplementary file 1 — ee3c00435_si_001.pdf [file ee3c00435_si_001.pdf]

Supporting Information

Feature engineering and supervised machine learning to forecast biogas production during municipal anaerobic co-digestion

Hunter W. Schroer, Craig L. Just*

Civil & Environmental Engineering, The University of Iowa, Iowa City, Iowa, 52242, United States

*Email: hunter-schroer@uiowa.edu

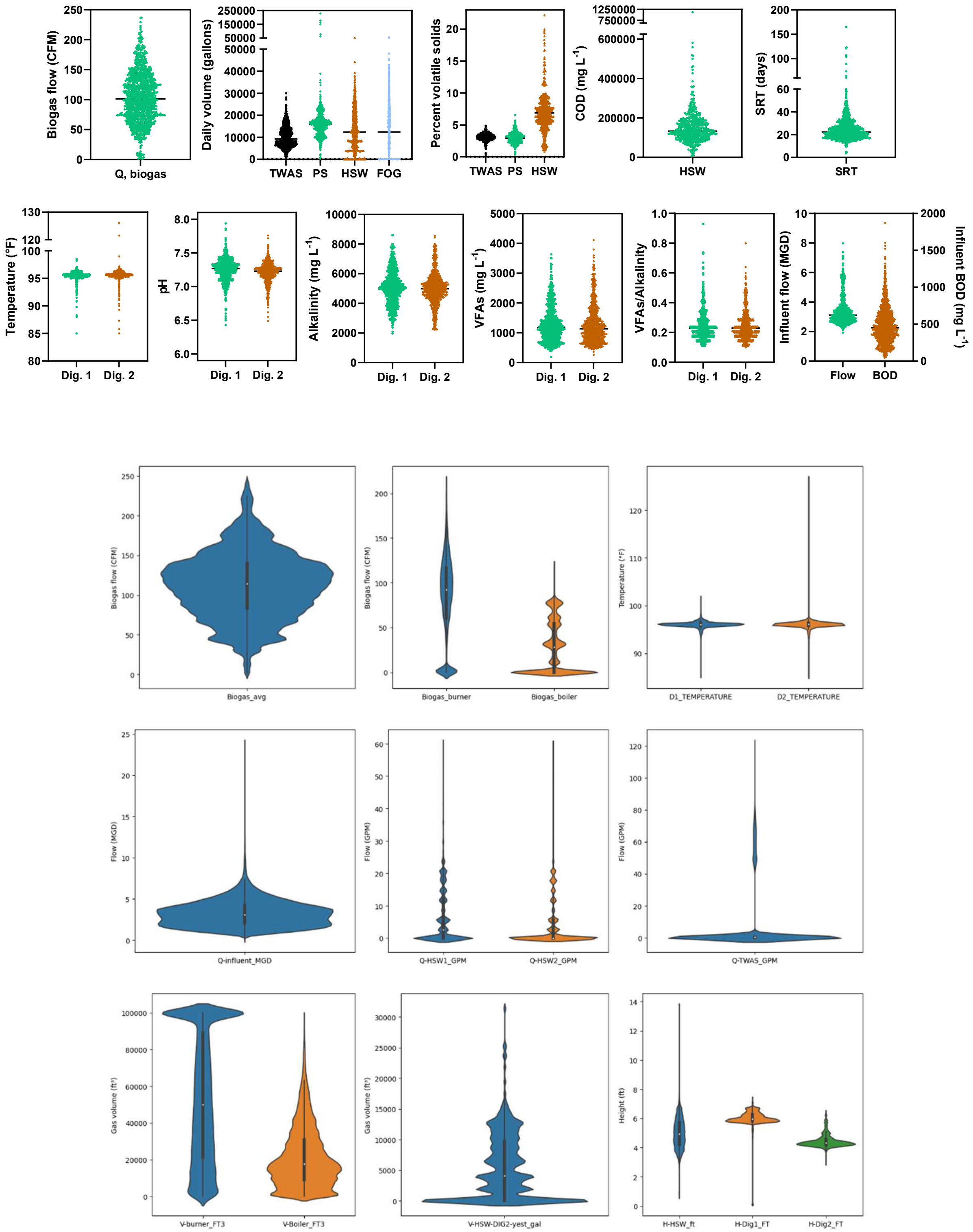

Figure S1. Distributions of data for the lab and SCADA datasets.

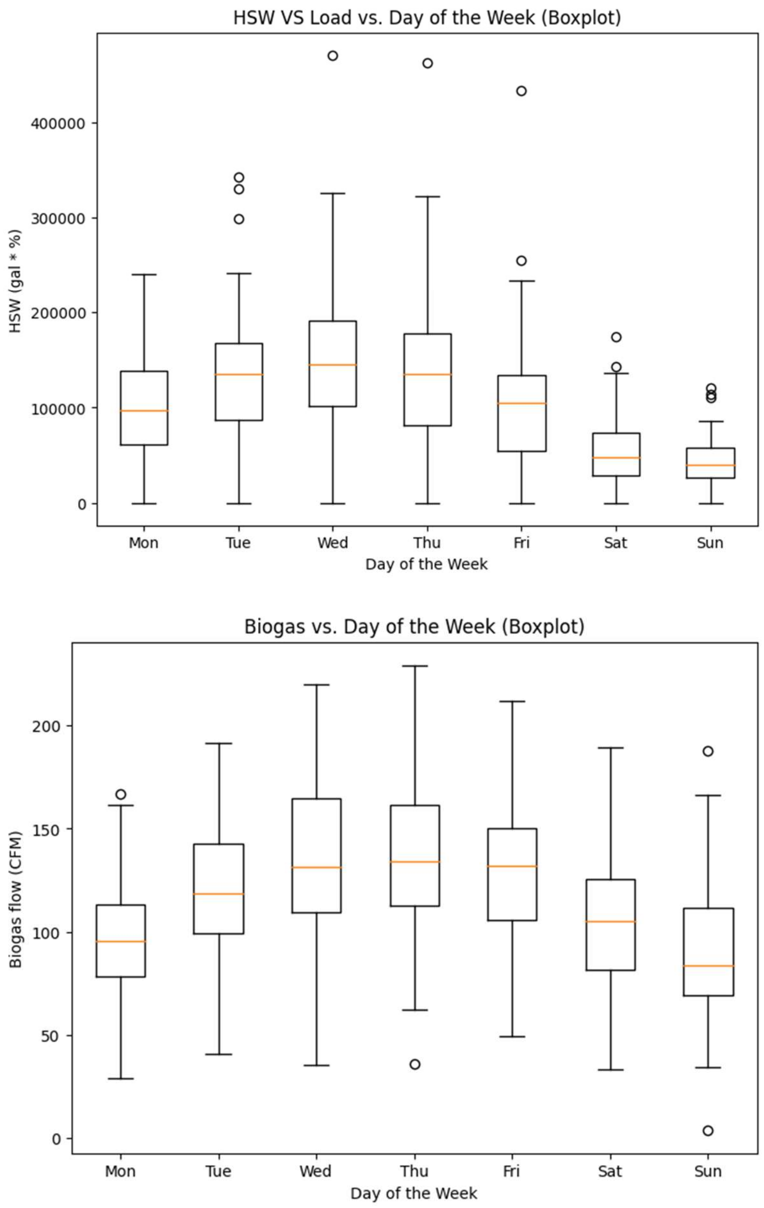

Figure S2. Weekly patterns of high strength waste VS loading (top) and biogas flow (bottom).

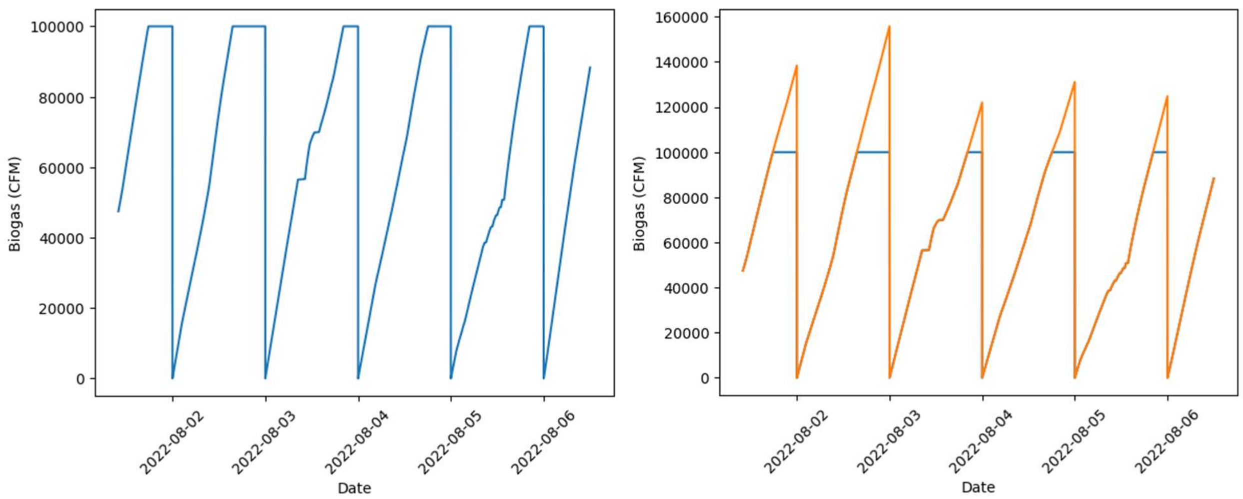

Figure S3. Volume of biogas to the burner today prior to (blue) and after (orange) re-calculation to prevent peak shaving at 100,000 cubic feet.

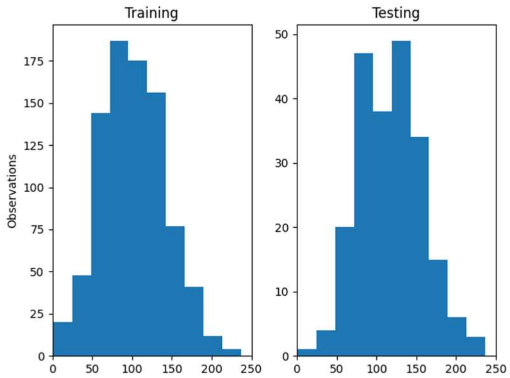

Figure S4. Histograms of the target variable, biogas flow over the next 24 hours, for the lab dataset with a training/testing split of 80/20%.

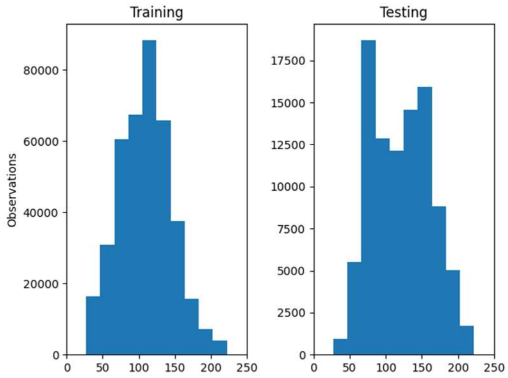

Figure S5. Histograms of the target variable, biogas flow over the next 24 hours, for the SCADA dataset with a training/testing split of 80/20%.

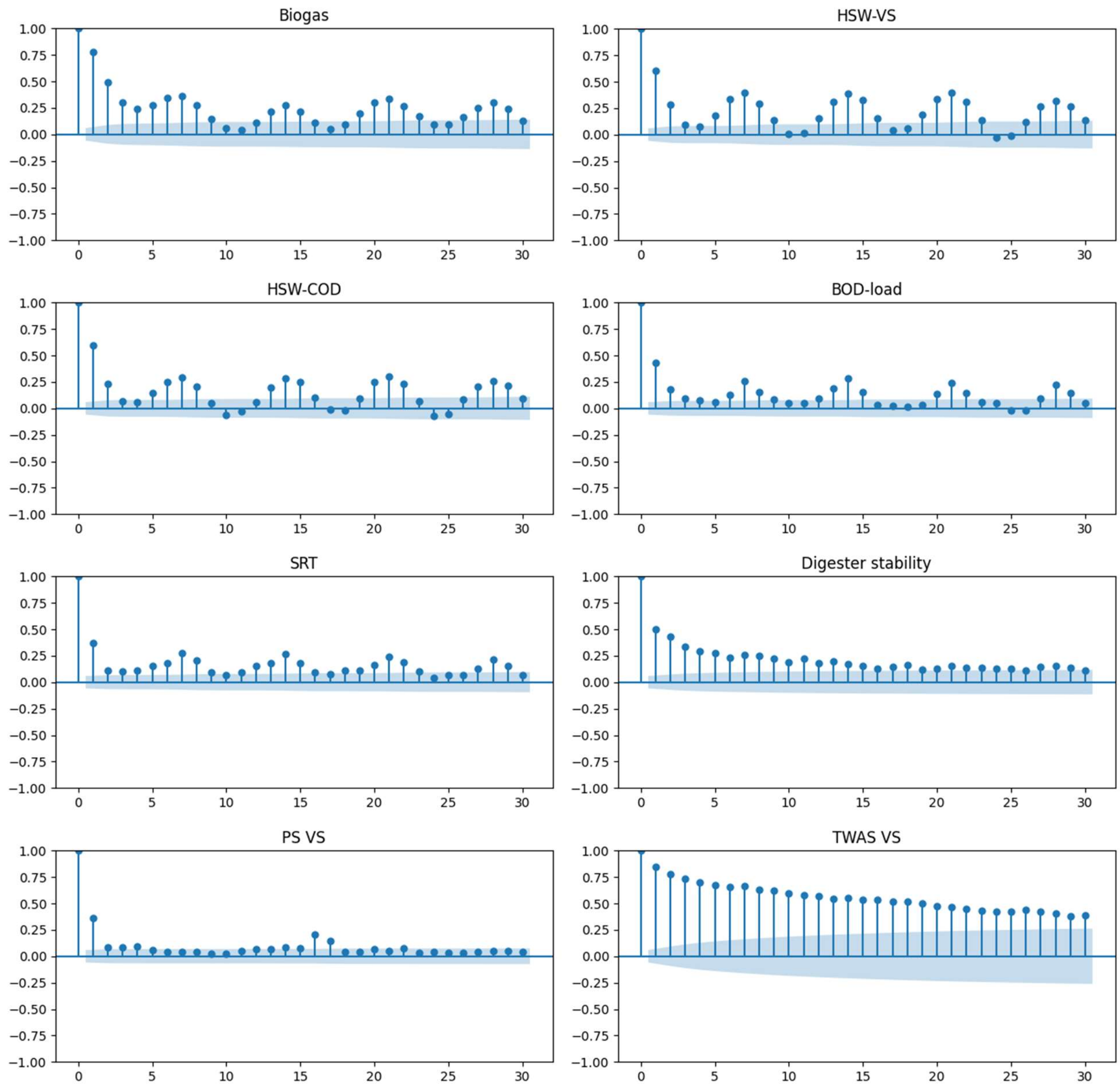

Figure S6. Autocorrelation plots for lab variables. The blue shaded region represents the area of no significant autocorrelation (α=0.05).

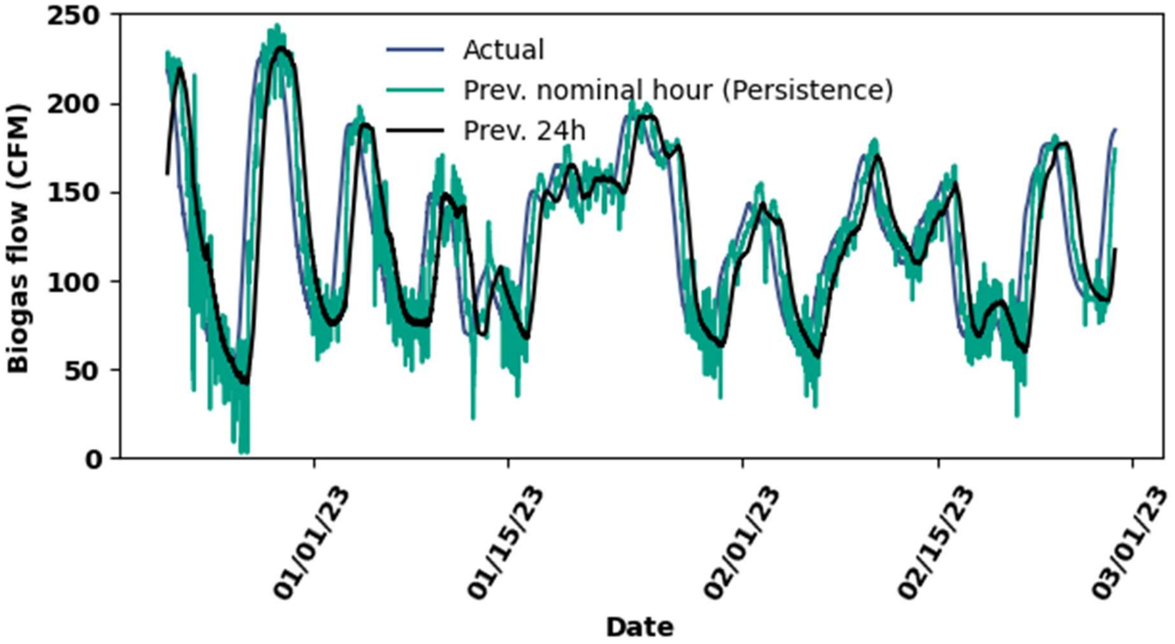

Baseline persistence model feature	RMSE (test), CFM	MAPE (test)	Adj. R^2 (test)	TeEI, CFM
Biogas previous nominal hour	27.1	17.9%	0.600	1.45
Biogas previous 24 hours	37.3	25.1%	0.242	9.75

Figure S7. Comparison of baseline, “persistence” models.

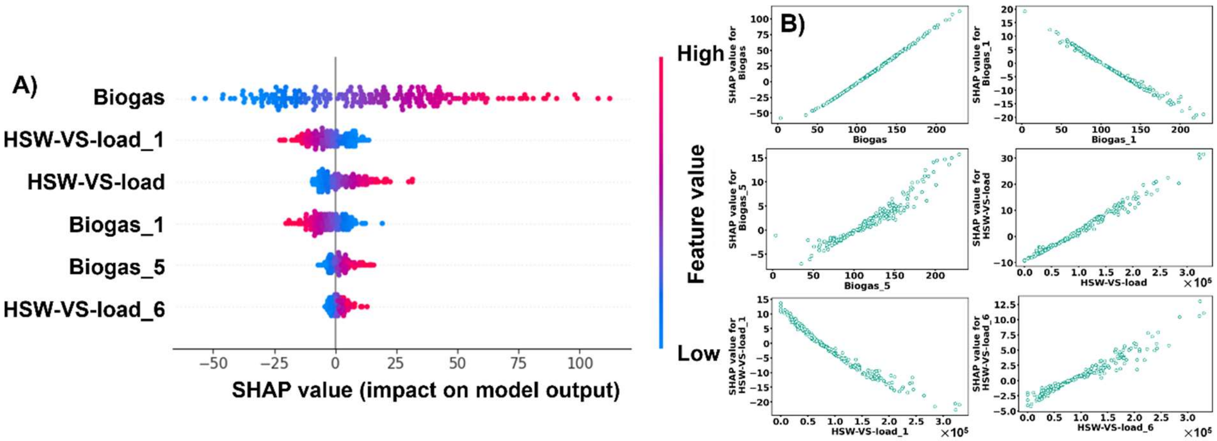

Figure S8. Lab dataset A) Shapely (SHAP) values as a function of feature value for each feature in the MLP model B) Individual dependence plots of the SHAP values as a function of the feature values for the MLP model.

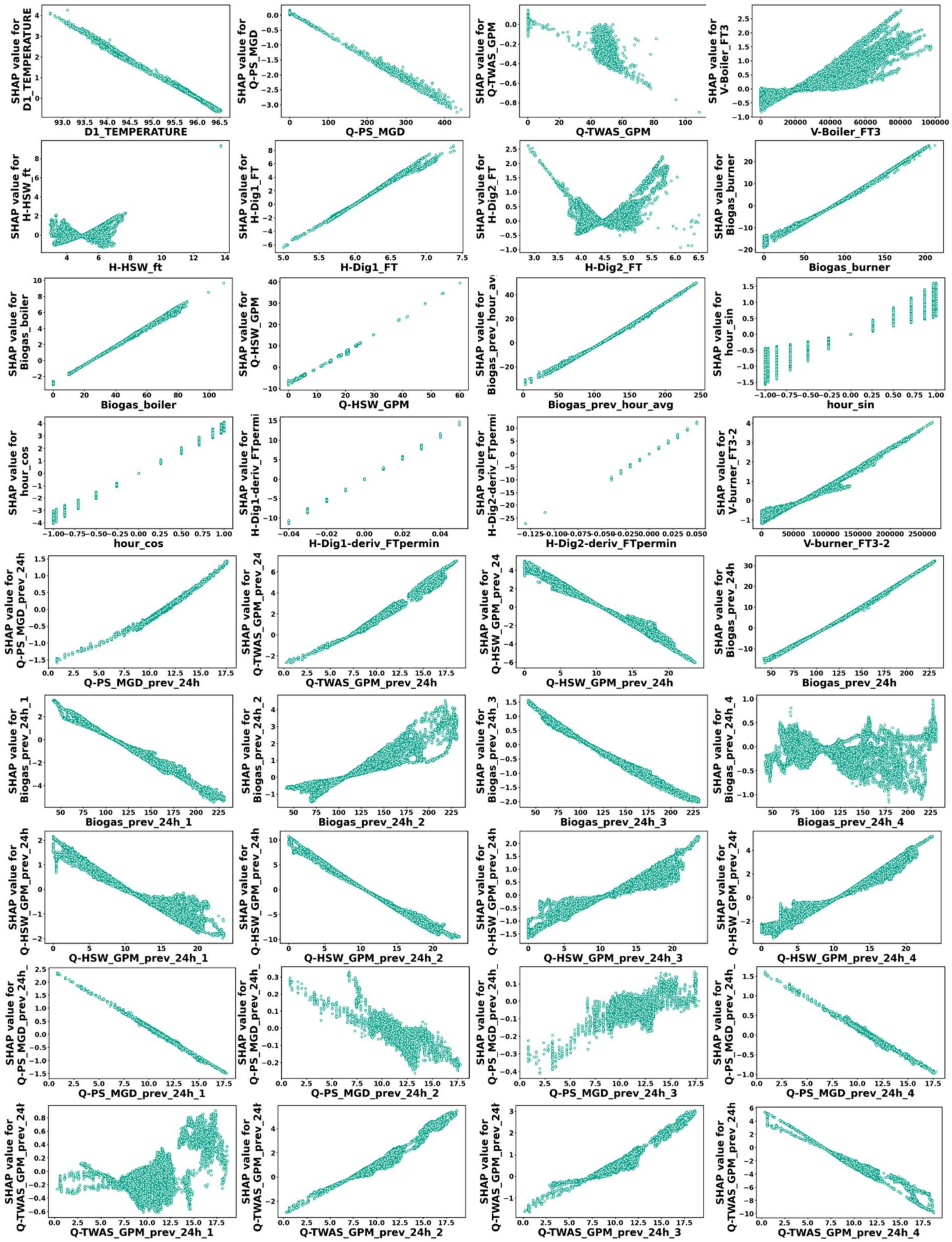

Figure S9. All SHAP dependence plots for the MLP model fit to the SCADA dataset

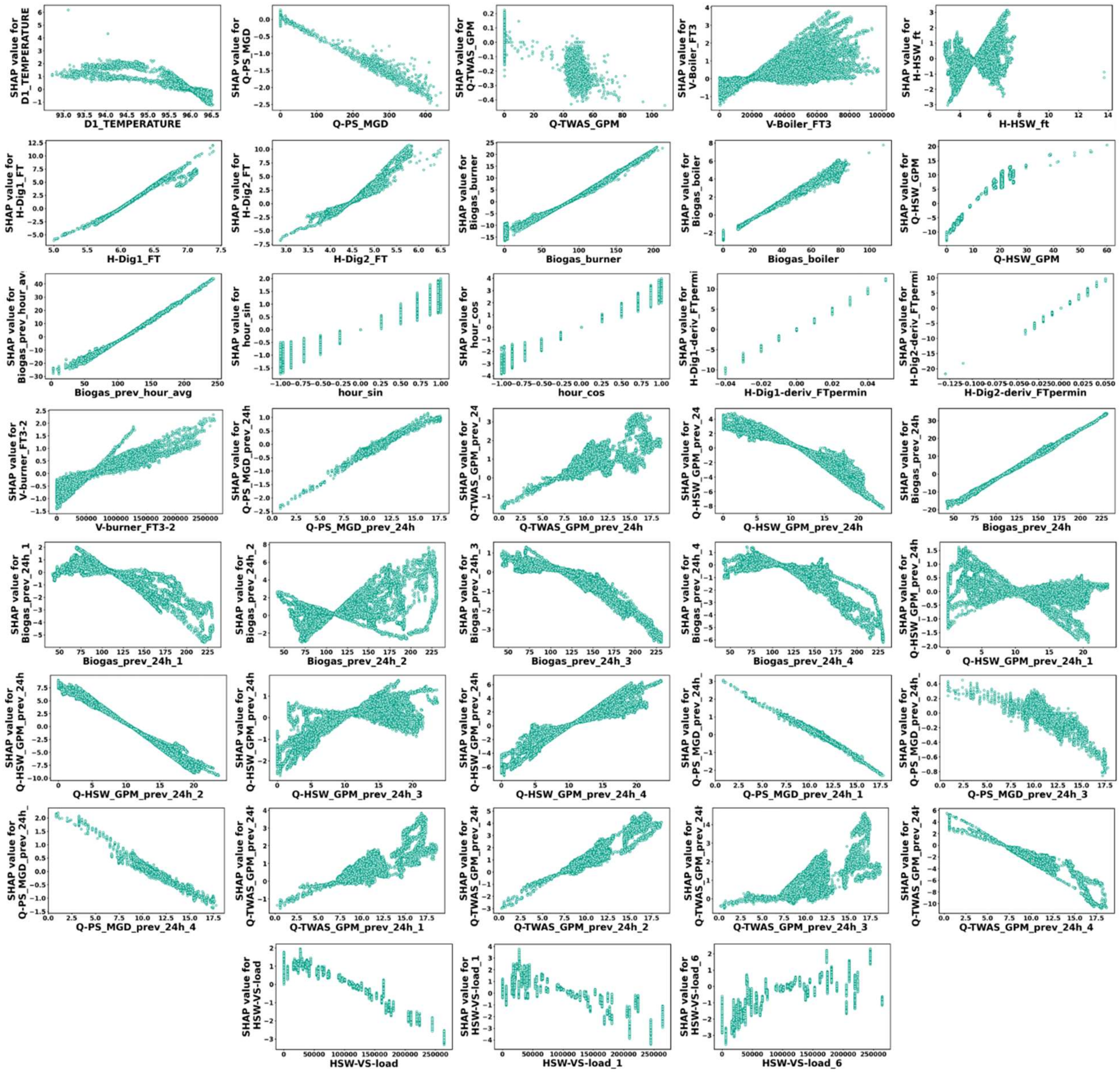

Figure S10. All SHAP dependence plots for the MLP model fit to the combined dataset

Table S1. Number of time points for each dataset as a function of forecast horizon for Figure 9.

Forecast horizon (d)	Dataset		
	Lab	SCADA	Combined
0.5	-	492,481	492,481
1	1,089	491,761	491,761
1.5	-	491,041	491,041
2	1,087	490,321	490,321
3	1,085	488,881	488,881

Table S2. MLP model hyperparameter tuning information. The initial hyperparameters were identified with *GridSearchCV*, and the number of hidden neurons and alpha values were further manually tuned to identify the model with the lowest TeEI.

Model hyper-parameter	**Optimal values** (search space)					
	Lab	Lab (on linear reg. error)	SCADA	SCADA (on linear reg. error)	Combined	Combined (on linear reg. error)
Number of hidden layers	**2** (1-2)	**2** (1-2)	**1** (1-2)	**1**	**2** (1-2)	**1**
Number of nuerons at each layer	**(20,7)** ([2-15] for 1 layer, [4-65,2-17] for 2 layers)	**(10,2)** ([2-15] for 1 layer, [4-65,2-17] for 2 layers)	**20** ([10-70] for 1 layer, [20-800,2-150 for 2 layers)	**60** (10-70)	**(400,100)** ([10-70] for 1 layer, [20-800,2-150 for 2 layers)	**40** (10-70)
Learning rate	**adaptive** (invscaling, adaptive)	**invscaling** (invscaling, adaptive)	**invscaling** (invscaling , adaptive)	**invscaling** (invscaling, adaptive)	**invscaling** (invscaling, adaptive)	**invscaling** (invscaling, adaptive)
Solver	**sgd** (lbfgs, sgd, adam)	**adam** (sgd, adam)	**sgd** (sgd, adam)	**sgd** (sgd, adam)	**sgd** (sgd, adam)	**sgd** (sgd, adam)
Strength of L2 regularization term (alpha)	**74** (0.0001-500)	**500** (0.0001-800)	**155** (0.01-500)	**500** (0.001-500)	**550** (0.0001-700)	**500** (0.001-500)
Activation function	**rectified linear unit function, "relu"**	**rectified linear unit function, "relu"**	**rectified linear unit function, "relu"**	**rectified linear unit function, "relu"**	**rectified linear unit function, "relu"**	**rectified linear unit function, "relu"**

Table S3. Features with significant (α = 0.05) coefficients in ridge multiple linear regression that were used for all models.

Lab (6)	SCADA (36)	Combined (38)
Biogas	Biogas_burner	Biogas_burner
Biogas (1-day lag)	Biogas_boiler	Biogas_boiler
Biogas (5-day lag)	Biogas_prev_hour_avg	Biogas_prev_hour_avg
HSW VS load	Biogas_prev_24h	Biogas_prev_24h
HSW VS load (1-day lag)	Biogas_prev_24h_1	Biogas_prev_24h_1
HSW VS load (6-day lag)	Biogas_prev_24h_2	Biogas_prev_24h_2
	Biogas_prev_24h_3	Biogas_prev_24h_3
	Biogas_prev_24h_4	Biogas_prev_24h_4
	Q-HSW_GPM	Q-HSW_GPM
	Q-HSW_GPM_prev_24h	Q-HSW_GPM_prev_24h
	Q-HSW_GPM_prev_24h_1	Q-HSW_GPM_prev_24h_1
	Q-HSW_GPM_prev_24h_2	Q-HSW_GPM_prev_24h_2
	Q-HSW_GPM_prev_24h_3	Q-HSW_GPM_prev_24h_3
	Q-HSW_GPM_prev_24h_4	Q-HSW_GPM_prev_24h_4
	Q-PS_MGD	Q-PS_MGD
	Q-PS_MGD_prev_24h	Q-PS_MGD_prev_24h
	Q-PS_MGD_prev_24h_1	Q-PS_MGD_prev_24h_1
	Q-PS_MGD_prev_24h_2	Q-PS_MGD_prev_24h_3
	Q-PS_MGD_prev_24h_3	Q-PS_MGD_prev_24h_4
	Q-PS_MGD_prev_24h_4	Q-TWAS_GPM
	Q-TWAS_GPM	Q-TWAS_GPM_prev_24h
	Q-TWAS_GPM_prev_24h	Q-TWAS_GPM_prev_24h_1
	Q-TWAS_GPM_prev_24h_1	Q-TWAS_GPM_prev_24h_2
	Q-TWAS_GPM_prev_24h_2	Q-TWAS_GPM_prev_24h_3
	Q-TWAS_GPM_prev_24h_3	Q-TWAS_GPM_prev_24h_4
	Q-TWAS_GPM_prev_24h_4	V-burner_FT3-2
	V-burner_FT3-2	Hour
	Hour	V-Boiler_FT3
	V-Boiler_FT3	H-HSW_ft
	H-HSW_ft	H-Dig1_FT
	H-Dig1_FT	H-Dig2_FT
	H-Dig2_FT	H-Dig1-deriv_FTpermin
	H-Dig1-deriv_FTpermin	H-Dig2-deriv_FTpermin
	H-Dig2-deriv_FTpermin	D1_TEMPERATURE
	D1_TEMPERATURE	HSW VS load
		HSW VS load (1-day lag)
		HSW VS load (6-day lag)

Table S4. Summary of results for each model.

Dataset (k, number of features used)	Model (K, number of parameters fit)	Performance metrics (test set)
Lab (6)	Ridge regression (8)	RMSE: 24.3
		MAPE: 0.276
		Adjusted R^2: 0.613
		AIC: 620
		TeEI: 3.03
	TPOT AutoML (undetermined)	RMSE: 24.5
		MAPE: 0.271
		Adjusted R^2: 0.607
		TeEI: 2.96
	MLP (267)	RMSE: 23.4
		MAPE: 0.265
		Adjusted R^2: 0.641
		AIC: 1,131
		TeEI: 2.57
	Ridge + MLP (275)	RMSE: 24.3
		MAPE: 0.276
		Adjusted R^2: 0.613
		AIC: 1,154
		TeEI: 3.02
SCADA (36)	Ridge regression (38)	RMSE: 20.9
		MAPE: 0.137
		Adjusted R^2: 0.761
		AIC: 259,925
		TeEI: 0.52
	TPOT AutoML (undetermined)	RMSE: 22.8
		MAPE: 0.150
		Adjusted R^2: 0.718
		TeEI: 0.71
	MLP (740)	RMSE: 20.3
		MAPE: 0.134
		Adjusted R^2: 0.776
		AIC: 258,648
		TeEI: 0.47
	Ridge + MLP (778)	RMSE: 21.0
		MAPE: 0.137
		Adjusted R^2: 0.761
		AIC: 261,508
		TeEI: 0.52
Combined (38)	Ridge regression (40)	RMSE: 21.3
		MAPE: 0.140
		Adjusted R^2: 0.752
		AIC: 261,535
		TeEI: 0.56
	TPOT AutoML (undetermined)	RMSE: 23.1
		MAPE: 0.157
		Adjusted R^2: 0.710
		TeEI: 0.80
	MLP (55,300)	RMSE: 20.3
		MAPE: 0.131
		Adjusted R^2: 0.775
		AIC: 367,910
		TeEI: 0.45
	Ridge + MLP (55,340)	RMSE: 21.3
		MAPE: 0.140
		Adjusted R^2: 0.753
		AIC: 372,080
		TeEI: 0.56

SCADA – supervisory control and data acquisition, MLP – multi-layer perceptron, RMSE – root mean squared error, MAPE – mean absolute percentage error, R^2 – coefficient of determination, AIC – Akaike information criterion, TeEI – testing error index, TPOT – tree-based Pipeline Optimization Tool, AutoML – automated machine learning.

Text S1: Digester stability calculation

We calculated a digester stability score for both digesters based on the method in Cook et al.[1] using digester temperature, pH, alkalinity, VFAs, and FOS/TAC. First, we transformed temperature to a normal distribution using a quantile transformer. Then we calculated and averaged a subscore for each variable, which was the stability score for each digester. Finally, we averaged the stability score from both digesters to give a single digester stability score. Each subscore was calculated as a value between zero and one based on threshold values as in Cook et al. For temperature, the subscore was 1 if within one standard deviation of the mean, 0.5 if within two standard deviations of the mean, and 0 otherwise. For pH, alkalinity, and VFAs, we matched the subscoring in Cook et al. For FOS/TAC ratio, the subscore was 0 if greater than 0.6, 0.5 if between 0.5 and 0.6, 0.75 if between 0.4 and 0.5, and 1 if below 0.4.

Text S2: TPOT AutoML output

The TPOT AutoML algorithm selects an optimal tree-based ML pipeline for the input dataset. For the lab dataset, the selected pipeline was a minimum to maximum scaler (scales all features to a range of 0-1) and a linear support vector regression. For the SCADA dataset, the selected pipeline was four consecutive multiple linear regressions. For the combined dataset, the selected pipeline involved feature selection prior to linear regression. Specifically, the pipeline nonsensically created two copies of the features and recombined them, then removed unimportant features based on F-regression scores. Finally, the selected features were fit with a linear support vector regression. Raw TPOT outputs are copied below:

LABS, raw TPOT output:

```
import numpy as np
import pandas as pd
from sklearn.model_selection import train_test_split
from sklearn.pipeline import make_pipeline
from sklearn.preprocessing import MinMaxScaler
from sklearn.svm import LinearSVR
from tpot.export_utils import set_param_recursive

# NOTE: Make sure that the outcome column is labeled 'target' in the data file
tpot_data = pd.read_csv('PATH/TO/DATA/FILE', sep='COLUMN_SEPARATOR',
dtype=np.float64)
features = tpot_data.drop('target', axis=1)
training_features, testing_features, training_target, testing_target = \
            train_test_split(features, tpot_data['target'], random_state=42)

# Average CV score on the training set was: -562.0504895488633
exported_pipeline = make_pipeline(
    MinMaxScaler(),
    LinearSVR(C=20.0, dual=True, epsilon=0.0001, loss="squared_epsilon_insensitive", tol=0.1)
)
# Fix random state for all the steps in exported pipeline
set_param_recursive(exported_pipeline.steps, 'random_state', 42)

exported_pipeline.fit(training_features, training_target)
results = exported_pipeline.predict(testing_features)
```

SCADA, raw TPOT output:

```
import numpy as np
import pandas as pd
from sklearn.linear_model import ElasticNetCV, LassoLarsCV, SGDRegressor
from sklearn.model_selection import train_test_split
from sklearn.pipeline import make_pipeline, make_union
from tpot.builtins import StackingEstimator
from tpot.export_utils import set_param_recursive

# NOTE: Make sure that the outcome column is labeled 'target' in the data file
tpot_data = pd.read_csv('PATH/TO/DATA/FILE', sep='COLUMN_SEPARATOR',
dtype=np.float64)
features = tpot_data.drop('target', axis=1)
training_features, testing_features, training_target, testing_target = \
            train_test_split(features, tpot_data['target'], random_state=1)

# Average CV score on the training set was: -329.51976744763334
exported_pipeline = make_pipeline(
    StackingEstimator(estimator=SGDRegressor(alpha=0.01, eta0=1.0, fit_intercept=False,
l1_ratio=0.25, learning_rate="invscaling", loss="huber", penalty="elasticnet", power_t=0.5)),
    StackingEstimator(estimator=ElasticNetCV(l1_ratio=0.8500000000000001, tol=0.1)),
    StackingEstimator(estimator=SGDRegressor(alpha=0.01, eta0=1.0, fit_intercept=False,
l1_ratio=0.25, learning_rate="invscaling", loss="huber", penalty="elasticnet", power_t=0.5)),
    LassoLarsCV(normalize=False)
)
# Fix random state for all the steps in exported pipeline
set_param_recursive(exported_pipeline.steps, 'random_state', 1)

exported_pipeline.fit(training_features, training_target)
results = exported_pipeline.predict(testing_features)
```

Combined, raw TPOT output:

```
import numpy as np
import pandas as pd
from sklearn.feature_selection import SelectPercentile, f_regression
from sklearn.model_selection import train_test_split
from sklearn.pipeline import make_pipeline, make_union
from sklearn.svm import LinearSVR
from tpot.builtins import StackingEstimator
from tpot.export_utils import set_param_recursive
from sklearn.preprocessing import FunctionTransformer
from copy import copy

# NOTE: Make sure that the outcome column is labeled 'target' in the data file
tpot_data = pd.read_csv('PATH/TO/DATA/FILE', sep='COLUMN_SEPARATOR',
dtype=np.float64)
features = tpot_data.drop('target', axis=1)
training_features, testing_features, training_target, testing_target = \
            train_test_split(features, tpot_data['target'], random_state=1)
```

```
# Average CV score on the training set was: -334.8915498690726
exported_pipeline = make_pipeline(
    make_union(
        FunctionTransformer(copy),
        FunctionTransformer(copy)
    ),
    SelectPercentile(score_func=f_regression, percentile=61),
    LinearSVR(C=0.001, dual=False, epsilon=0.1, loss="squared_epsilon_insensitive", tol=1e-05)
)
# Fix random state for all the steps in exported pipeline
set_param_recursive(exported_pipeline.steps, 'random_state', 1)

exported_pipeline.fit(training_features, training_target)
results = exported_pipeline.predict(testing_features)
```

Reference

1 Cook, S. M., Skerlos, S. J., Raskin, L. & Love, N. G. A stability assessment tool for anaerobic codigestion. *Water Research* **112**, 19-28 (2017). https://doi.org:https://doi.org/10.1016/j.watres.2017.01.027
